# High-Mobility Group Box Protein 3 (HMGB3) Facilitates DNA Interstrand Crosslink Processing and Double-Strand Break Repair in Human Cells

**DOI:** 10.3390/genes16091044

**Published:** 2025-09-04

**Authors:** Jillian Dangerfield, Anirban Mukherjee, Wade Reh, Anna Battenhouse, Karen M. Vasquez

**Affiliations:** 1 Division of Pharmacology and Toxicology, Dell Pediatric Research Institute, College of Pharmacy, The University of Texas at Austin, 1400 Barbara Jordan Boulevard, Austin, TX 78723, USA; 2 Refeyn Inc., 21 Hickory Drive, Waltham, MA 02451, USA; anirban.mukherjee@refeyn.com; 3 Research and Extension, Kansas State University, Manhattan, KS 66506, USA; 4 Center for Biomedical Research (CBRS) Bioinformatics Consulting Group (BCG), The University of Texas at Austin, Austin, TX 78723, USA

**Keywords:** high-mobility group box protein 3 (HMGB3), DNA damage repair, DNA interstrand crosslink (ICL), DNA double-strand break (DSB) repair, cancer

## Abstract

**Background/Objectives**: DNA-damaging agents can contribute to genetic instability, and such agents are often used in cancer chemotherapeutic regimens due to their cytotoxicity. Thus, understanding the mechanisms involved in DNA damage processing can not only enhance our knowledge of basic DNA repair mechanisms but may also be used to develop improved chemotherapeutic strategies to treat cancer. The high-mobility group box protein 1 (HMGB1) is a known nucleotide excision repair (NER) cofactor, and its family member HMGB3 has been implicated in chemoresistance in ovarian cancer. Here, we aim to understand the potential role(s) of HMGB3 in processing DNA damage. **Methods**: A potential role in NER was investigated using HMGB3 knockout human cell lines in response to UV damage. Subsequently, potential roles in DNA interstrand crosslink (ICL) and DNA double-strand break (DSB) repair were investigated using mutagenesis assays, metaphase spreads, foci formation, a variety of DNA repair assays, and TagSeq analyses in human cells. **Results**: Interestingly, unlike HMGB1, HMGB3 does not appear to play a role in NER. We found evidence to suggest that HMGB3 is involved in the processing of both DSBs and ICLs in human cells. **Conclusions**: These novel results elucidate a role for HMGB3 in DNA damage repair and, surprisingly, also indicate a distinct role of HMGB3 in DNA damage repair from that of HMGB1. These findings advance our understanding of the role of HMGB3 in chemotherapeutic drug resistance and as a target for new chemotherapeutic strategies in the treatment of cancer.

## 1. Introduction

DNA damage occurs from both endogenous and exogenous sources, and error-free repair is essential to ensure genome integrity. Endogenous DNA damage forms at a rate of around 10^4^ to 10^5^ lesions per cell per day, simply from byproducts arising from normal cellular metabolic processes [1,2,3]. There is also a wide variety of exogenous agents that can induce DNA damage, such as UV irradiation from the sun, as well as toxic and/or chemotherapeutic drugs [1,2]. DNA damage left unrepaired and/or processed in an error-generating fashion can lead to genomic instability and subsequently the development of cancer or other diseases [4]. However, the DNA-damaging effects of chemotherapeutic drugs can be utilized to kill cancer cells as a form of treatment, although the development of resistance to these drugs is a common obstacle that continues to be limiting [5,6]. Thus, elucidating the mechanisms of DNA damage repair informs our understanding of canonical DNA repair to maintain genome integrity as well as chemotherapeutic treatment applications.

High-mobility group box (HMGB) proteins are nuclear non-histone architectural proteins. We have previously found that one of the members of this family of proteins, HMGB1, is involved in the repair of both DNA intrastrand crosslinks and DNA interstrand crosslinks (ICLs) [7,8,9,10]. DNA intrastrand crosslinks are a type of damage in which a covalent bond is formed between two bases on the same strand of DNA, while ICLs form covalent bonds between two bases on opposite DNA strands. DNA intrastrand crosslinks are often repaired by the nucleotide excision repair (NER) [11] mechanism, and ICLs are processed by proteins from a number of repair pathways, including, but not limited to, NER [12]. NER has been shown to be involved in processing ICLs, particularly in G_0_/G_1_ cells, while DNA double-strand break (DSB) repair mechanisms are more prevalent during G_2_, as DSBs are often intermediates of ICL processing during replication [12,13,14,15]. These lesions are highly cytotoxic, and many chemotherapeutic agents form ICLs as the main cytotoxic lesion in cancer chemotherapy [13,15].

The HMGB family comprises a group of architectural proteins first discovered because of their high mobility on polyacrylamide gels observed in purified calf thymus samples [16]. These proteins bind to and bend non-canonical DNA structures in a non-sequence-specific manner, impacting DNA structure and flexibility [17,18,19]. This function is facilitated by the structure of these proteins, which consists of two box domains and a C-terminal acidic tail, which are features shared by three of the four members of this protein family (HMGB1, HMGB2, and HMGB3) [18]. The fourth member of this family, HMGB4, does not contain the C-terminal acidic tail [18] and is not discussed further here.

HMGB1, HMGB2, and HMGB3 share 80% sequence homology, yet they appear to play distinct biological roles [18,20,21,22,23]. Some of these differences can be explained by variation in expression patterns. HMGB1 is ubiquitously expressed and is the second most abundant protein, after histones, in the nucleus [18]. HMGB1 also has a cytokine function in the cell [24,25,26,27], and the absence of HMGB1 is lethal in knockout mice due to hypoglycemia [20]. HMGB2 and HMGB3 knockout in mice does not confer lethality; rather, the absence of each of these proteins disrupts spermatogenesis [21] and hematopoietic stem cell differentiation [22,23], respectively. Accordingly, HMGB2 is expressed in the thymus and testes, HMGB3 is expressed in adult bone marrow, and both are expressed during development [21,22,28]. Additionally, HMGB3 is an X-linked protein; hence, a single copy is expressed [22].

Of the three proteins, the most work has been done to understand the cellular functions of HMGB1. This includes its roles in DNA damage repair; for example, we have identified it as an NER cofactor [8,9,10,27,29]. NER is the dedicated DNA repair pathway for processing the intrastrand crosslinks formed by UV irradiation [11]. We have previously found that HMGB1 contributes to increased cell survival in response to UVC irradiation, promotes error-free repair of UVC-induced DNA damage, and facilitates the removal of both types of UVC-induced intrastrand crosslinks, 6-4 photoproducts (6-4 PPs) and cyclobutene pyrimidine dimers (CPDs) in mouse embryonic fibroblasts [8]. A proposed mechanism for the role of HMGB1 in DNA damage repair is that it binds to damaged DNA (a non-canonical DNA structure) and then bends the DNA at the site of the damage [8]. This, in turn, facilitates recruitment of NER proteins, which recognize damage by the distortion of the DNA helix [8,11]. This is especially relevant in the context of chromatin, where distorted structures caused by DNA damage may be more difficult to detect without the aid of an architectural protein such as HMGB1 [29]. We have also provided additional supporting evidence of HMGB1 in structure-associated DNA damage repair processing, where HMGB1 promotes excess negative supercoiling of damaged DNA in cell extracts [10] and chromatin remodeling in vitro [8].

Not only has HMGB1 been implicated in NER processing of canonical UV irradiation-induced DNA lesions, but it has also been found to be involved in processing of complex ICLs [9,10], which can also induce DNA helical distortions [15]. HMGB1 has been found to bind triplex-directed psoralen ICLs (pTFO-ICLs) with the NER complexes, XPA-RPA, and XPC-RAD23B in vitro [9], and contributes to NER-mediated error-free repair of pTFO-directed ICLs [10].

Despite the striking structural and sequence similarity of HMGB3 and HMGB1, this third member of the HMGB protein family has not been well studied for its potential role in DNA repair. However, we have identified a role for HMGB3 in resistance to cisplatin, a DNA-damaging chemotherapeutic agent, in human ovarian cancer cells [30], suggesting that HMGB3 might be involved in DNA damage repair. Furthermore, similar sensitization of cancer cells to other DNA-damaging chemotherapeutic agents through HMGB3 depletion has been observed in other cancer types [31,32,33,34], and HMGB3 has been shown to be a potential biomarker for a variety of cancers, where high expression of HMGB3 is associated with poor patient prognosis [35]. Therefore, understanding the role(s) of HMGB3 in cells can shed further light on the mechanisms behind these observations and provide information on HMGB3 as a potential target in cancer treatment [36].

The question we asked here is whether HMGB3, in addition to its transcriptional regulation of *ATR* [30], is involved in the processing of DNA damage by modulating DNA repair by NER, similar to HMGB1, and/or DSB and ICL processing. We found that HMGB3 appears to play a biologically relevant role in modulating ICL and DSB repair, while it appears to have a minimal role in NER. Additionally, we found evidence to support a role for HMGB3 in cisplatin resistance and in the modulation of the expression of many genes. These findings reveal new information on the functions of HMGB3, specifically with respect to DNA damage repair, and provide additional evidence for the mechanisms of its involvement in resistance to chemotherapy. The results from these studies have important implications for future work combating resistance to DNA-damaging chemotherapeutic agents and ultimately improving strategies to treat cancer.

## 2. Results

### 2.1. HMGB3, Unlike HMGB1, Does Not Play a Substantial Role in Nucleotide Excision Repair (NER)

**Figure 1:**
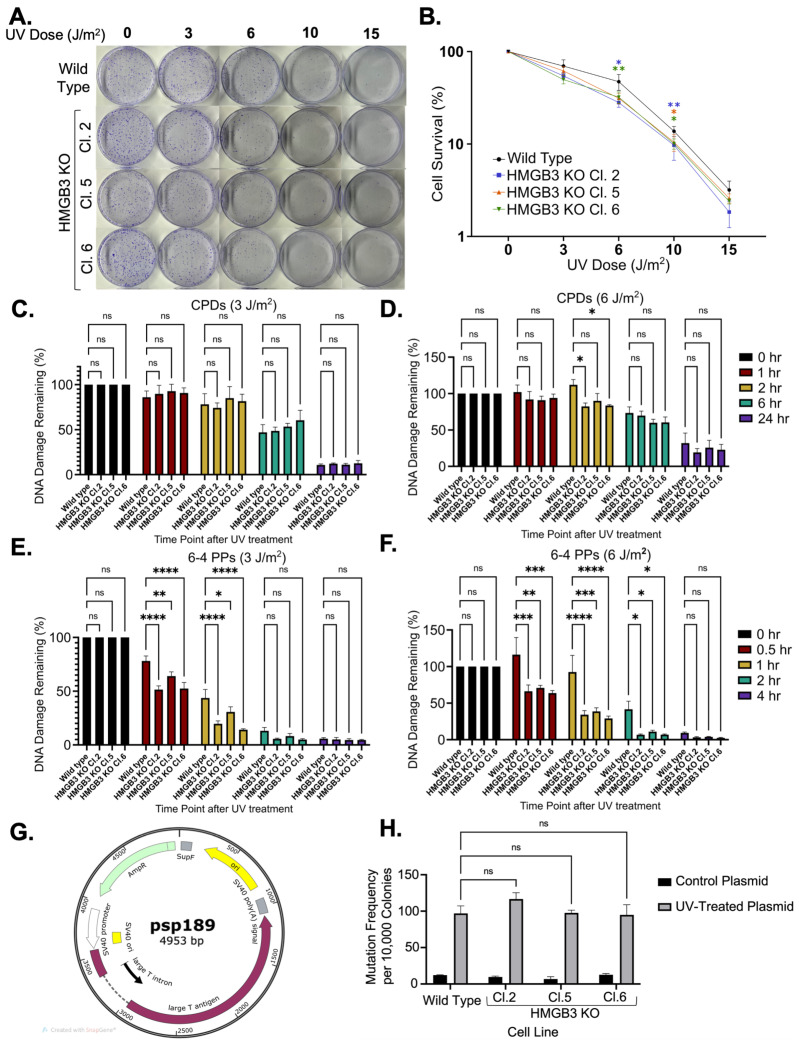
HMGB3 knockout minimally decreases cell survival in response to UVC irradiation in human U2OS cell lines. (**A**) Clonogenic assays and (**B**) quantification of clonogenic assays (N = 4), determining the effect of HMGB3 knockout on cell survival in response to 0 J/m^2^, 3 J/m^2^, 6 J/m^2^, 10 J/m^2^, and 15 J/m^2^ UVC treatment. At 3 J/m^2^ and 6 J/m^2^, the cell survival in the HMGB3 KO clones is significantly (*p* < 0.05) less than that of the wild-type cell line. At 10 J/m^2^ and 15 J/m^2^, the cell survival in the HMGB3 KO clones is not significantly different than that of the wild-type cell line. (**C**) Slot blot assays (N = 3) showing the effect of HMGB3 knockout on UVC-induced DNA damage removal. No significant difference was observed between the HMGB3 KO clones compared to the wild-type cell line in CPD removal following 3 J/m^2^ at any time point. (**D**) Following 6 J/m^2^, there was a significant (*p* < 0.05) increase in CPD removal at 2 h post-UVC treatment in the HMGB3 KO clones compared to the wild-type cell line. (**E**) There was a significant increase in 6-4 PP removal in the HMGB3 KO clones as compared to the wild-type cell line at both 3 J/m^2^ at 0.5 h and 1 h post-UVC treatment and following (**F**) 6 J/m^2^ at 0.5 h, 1 h, and 2 h post-UVC treatment. (**G**) Schematic representation depicting the mutation reporter, psp189, which contains an SV40 origin of replication, used in the mutagenesis assays. Briefly, plasmids were irradiated with UVC (350 J/m^2^), and UVC-induced damage in the *SupF* region resulted in white colonies for the mutagenesis assays, as previously described [8]. Blue-white screening was conducted in the same manner that mutagenesis assays with pTFO-ICLs have been conducted in previous publications [10,37]. (**H**) Mutagenesis assays (N = 3) utilizing blue-white screening of the transfected UVC-treated mutation reporters, showing no significant differences in mutation frequencies between the wild-type and HMGB3 knockout U2OS cell lines; error bars = SEM. ns = non-significant; *, *p* < 0.5; **, *p* < 0.01; ***, *p* < 0.001, **** *p* < 0.0001.

Experiments using UV-induced DNA damage were conducted in HMGB3 knockout human U2OS cells to investigate if HMGB3, like HMGB1, functions as an NER cofactor. HMGB3 knockout was verified through Sanger sequencing and Western blot analysis (Supplementary Figure S1). Clonogenic assays following UV irradiation at 3, 6, 10, and 15 J/m^2^ revealed that HMGB3 knockout significantly reduced cell survival following 3 J/m^2^ and 6 J/m^2^ of UVC treatment, but no significant differences were observed following irradiation with 10 J/m^2^ or 15 J/m^2^ of UVC (Figure 1A,B). Slot blot assays were used to determine DNA damage removal in the presence or absence of HMGB3, where both CPDs and 6-4 PPs were measured using respective DNA damage-specific antibodies following both 3 J/m^2^ and 6 J/m^2^ of UVC irradiation. Slot blot analysis revealed insignificant differences in CPD removal between HMGB3 knockout cells and WT cells, except at 2 h post-6 J/m^2^ of UVC irradiation, where HMGB3 knockout clones had slightly more lesion removal than the WT cells (Figure 1C,D; Supplementary Figure S2). More 6-4 PP were removed in HMGB3 knockout cells compared to WT cells following 3 J/m^2^ and 6 J/m^2^ at earlier timepoints (0.5 to 1 h- and 0.5 to 2 h-post UVC treatment, respectively), but not at later timepoints (2 to 4 h- and 4 h-post UVC treatment, respectively; Figure 1E,F).

Next, assays were conducted to assess the mutagenic processing of UV-induced DNA damage. A mutation reporter (psp189) containing a *SupF* mutation-reporter gene was used for blue-white screening to determine mutation frequencies and spectra (Figure 1G). No significant differences in mutagenic processing of UVC-induced lesions between HMGB3 knockout cells and WT cells were observed (Figure 1H), and no apparent differences were observed between HMGB3 knockout cells and WT cells in the mutation spectra analyses (Table 1), which comprised >90% point mutations in both WT and HMGB3 KO U2OS cells. These data suggest that, unlike HMGB1 [8,10], HMGB3 does not appear to play a substantial role in error-free UV-induced lesion processing in human cells.

### 2.2. HMGB3 Depletion Increases Mutation Frequencies, Large Deletions, and Chromosomal Aberrations in Response to ICLs

**Figure 2:**
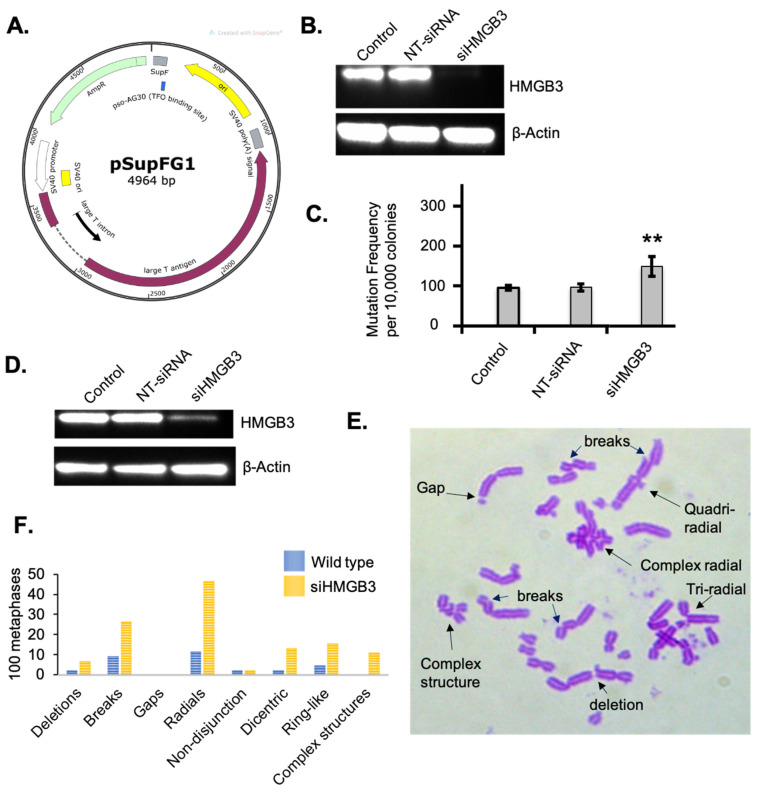
HMGB3 depletion in human U2OS cells results in mutagenic TFO-directed ICL processing and increases chromosomal anomalies. (**A**) Schematic representation of the pSupFG1 mutation reporter and the location of the bound triplex-forming oligonucleotide (TFO) with the position of the psoralen interstrand crosslink (ICL), as previously described [10,37]. Briefly, pTFO-ICLs are directed to the *SupF* region of the pSupFG1 plasmid, containing an SV40 origin of replication, resulting in white colonies that are quantified in number and sequenced during blue-white screening. (**B**) A representative western blot showing depletion of HMGB3 in human U2OS cells after treating with HMGB3 siRNA for the mutagenesis assays in Figure (**C**). (**C**) Mutation frequencies generated from the pSupFG1 mutation reporter in U2OS cells with or without TFO-directed ICLs as a function of siRNA-mediated HMGB3 depletion (N = 3). NT-siRNA = non-targeted siRNA treated; siHMGB3 = HMGB3 siRNA treated; error bars = ±SD. (**D**) A representative Western blot assessing HMGB3 depletion after siRNA treatment for the metaphase spreads. (**E**) A representative metaphase spread from U2OS cells following HMGB3 depletion and induction of psoralen ICLs, showing different predominant anomalies observed. Briefly, cells were treated with 10^−9^ M psoralen and 1.8 J/m^2^ UVA (365 nm) and were arrested with 10 μg/mL KaryoMAX Colcemid (ThermoFisher, Waltham, MA, USA) for 2 h before collection. (**F**) One hundred metaphase spreads from three separate experiments showing the different chromosomal anomalies that occurred in wild-type and HMGB3-depleted U2OS cells following psoralen and UVA treatment. **, *p* < 0.01.

In order to investigate the mutagenic repair of ICLs as a function of HMGB3 depletion, a mutagenesis assay using a *supF* mutation-reporter containing site-specific pTFO-ICLs was conducted in human U2OS cells (Figure 2A). HMGB3 depletion, assessed by immunoblotting (Figure 2B), increased the mutagenic repair of the pTFO-ICLs ~1.5-fold compared to WT cells (Figure 2C). The mutation spectra revealed that the mutations induced in the HMGB3-depleted cells were comprised of fewer point mutations (31.5%) than the WT cells (100%), a higher proportion of deletions than in the WT cells (36.3% with HMGB3-depletion compared to 0% in the WT cells), with additional mutations consisting of ~21% and ~10% insertions and single nucleotide deletions, respectively, which were not observed in the WT cells (Table 2). Detailed sequencing results can be found in Supplementary Figure S3.

Next, metaphase spread experiments were conducted to investigate chromosomal stability as a function of HMGB3 depletion in response to psoralen ICLs. In HMGB3-depleted human U2OS cells (Figure 2D), there was a substantial increase in chromosomal aberrations following treatment with psoralen plus UVA irradiation to induce ICLs (Figure 2E,F). The number of deletions in the HMGB3-depleted samples increased ~3-fold, DNA breaks increased ~2.8-fold, radials increased ~4-fold, dicentric chromosomes increased ~5.7-fold, ring-like chromosomes increased ~3.3-fold, and complex structures increased ~4.6-fold, as compared to WT U2OS cells. These results indicate that depletion of HMGB3 may result in increased deletions while processing complex DNA lesions such as ICLs, which is consistent with an increase in chromosomal radial formation and other chromosomal aberrations associated with defects in DSB processing.

### 2.3. HMGB3 Depletion Decreases Cell Survival and Reduces the Efficiency of DSB-Repair Pathways in Response to DSBs

**Figure 3:**
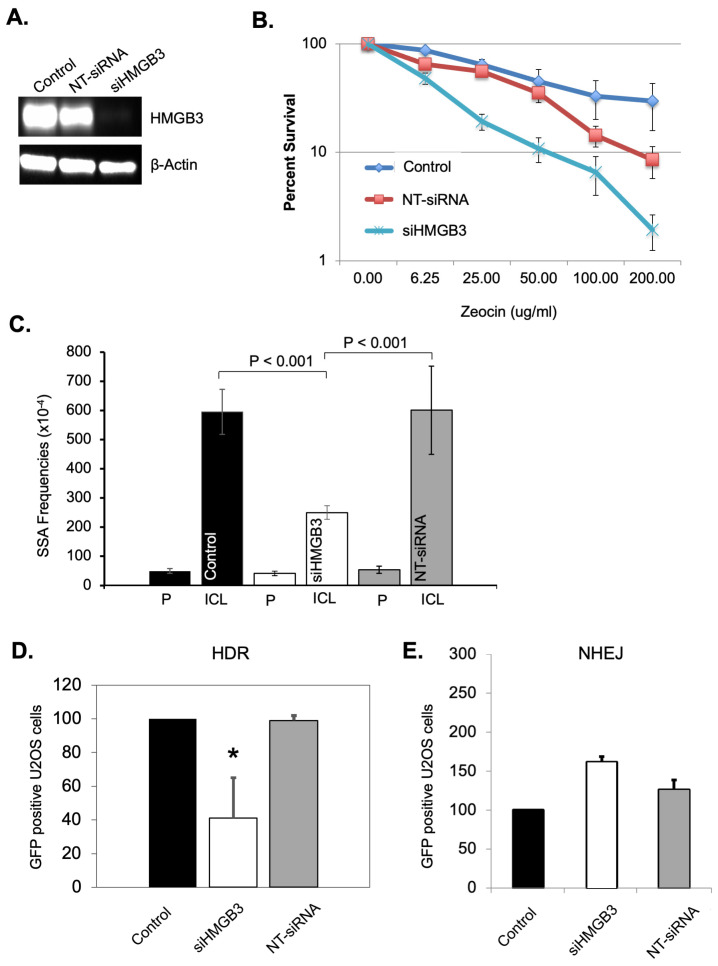
HMGB3 depletion impacts DNA double-strand break repair processes. (**A**) A representative western blot assessing HMGB3 depletion after siRNA treatment in U2OS cells. (**B**) Clonogenic survival assays as a function of siRNA-mediated HMGB3 depletion and Zeocin treatment in human U2OS cells (N = 3). Zeocin-treated U2OS cells showed lower survival following siRNA-mediated HMGB3 depletion. (**C**) SiRNA-mediated HMGB3 depletion reduces single-strand annealing (SSA) events in U2OS cells as observed by blue-white screening induced by TFO-directed ICLs (N = 3). A description of the SSA assay can be found in Liu, Nairn, and Vasquez [39], with the associated schematic in Figure 1B [39]. (**D**) GFP reporter assays in U2OS cells showed fewer homology-directed repair (HDR) events upon HMGB3 depletion following ISceI-mediated DSBs (N = 3). A schematic and description of the homology-directed repair reporter system can be found in Figure 1B of Pierce et al. [40], and a similar description can be found in Figure 1 of Cavallo et al. [41]. (**E**) GFP reporter assays showed HMGB3 depletion does not significantly modify NHEJ events following ISceI-induced DNA DSBs in U2OS cells (N = 3). A schematic and description of the assay can be found in Ogiwara et al. [42]; error bars = ±SD. *, *p* < 0.5.

One of the mechanisms employed in ICL repair involves DSB repair proteins/pathways; therefore, a series of DSB repair assays was conducted to investigate the role of HMGB3 in DSB processing. Clonogenic survival of U2OS cells was measured after treating the cells with 200 μg/mL of zeocin in the presence or absence of HMGB3. HMGB3 depletion was achieved using siRNA (Figure 3A). HMGB3 depletion substantially sensitized the U2OS cells to zeocin treatment compared to mock siRNA-treated and non-siRNA-treated cells (Figure 3B). As zeocin is a DSB-inducing agent, these results may indicate the involvement of HMGB3 in modulating DSB repair processes. Subsequently, a possible role of HMGB3 in DSB processing was studied using three different reporter systems. Identifying the role that HMGB3 may play in one or multiple DSB repair pathways, which differ in mechanism and in levels of error-free and error-generating outcomes, can elucidate the mechanisms underlying the mutation spectra data collected in response to ICL-induced DNA damage (see Table 2 and Supplementary Figure S3). Single-strand annealing (SSA) is an error-prone DSB repair pathway that resects DNA ends to align with homologous sequences during repair, which often leads to large deletions [38]. Homologous recombination is generally an error-free process, using an undamaged template to repair the damage, while NHEJ, a pathway without end resection, can be prone to errors [38]. An SSA assay revealed that HMGB3 depletion in U2OS cells led to a significant (*p* < 0.001) ~60% decrease in SSA events as compared to untreated and control siRNA-treated samples (Figure 3C). A DR-GFP-reporter assay was then utilized to detect homology-directed repair (HDR) events and revealed that HMGB3 depletion significantly reduced these events by ~60% (*p* < 0.05) in U2OS cells (Figure 3D). We also used a similar reporter assay to measure NHEJ frequency as a function of HMGB3, where HMGB3 depletion did not appear to significantly impact NHEJ events in U2OS cells (Figure 3E). These results indicate that HMGB3 is associated with SSA and HDR, but not NHEJ, in the processing of DSBs.

### 2.4. HMGB3 Forms Foci Following Cisplatin Treatment and HMGB3 Depletion Delays Cisplatin-Induced DNA Damage Repair

**Figure 4:**
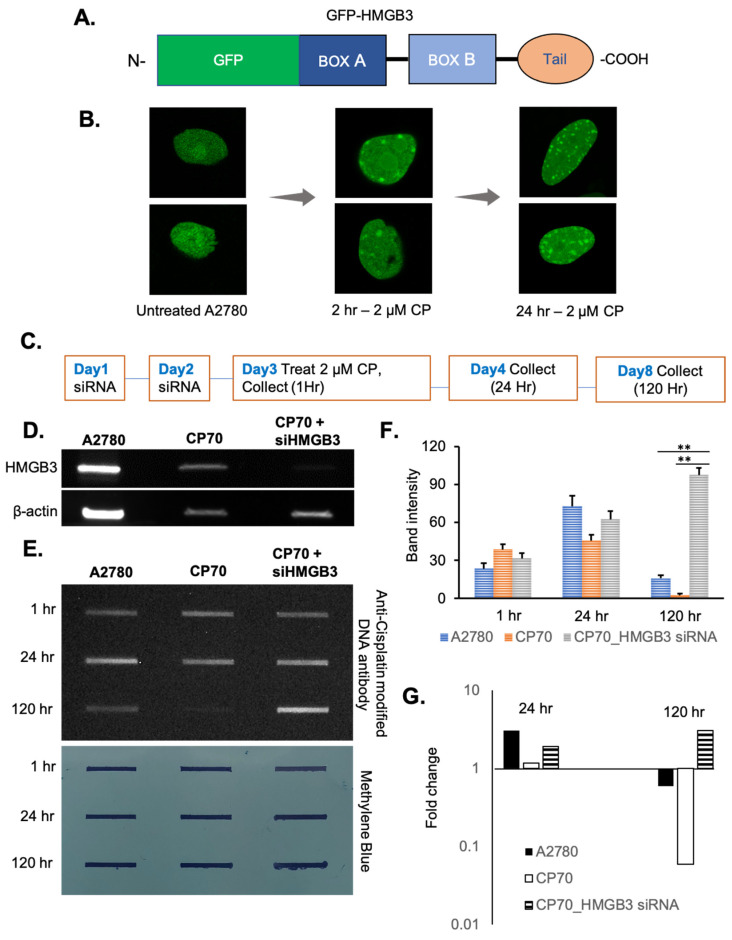
HMGB3 forms foci upon cisplatin treatment in A2780 human ovarian cancer cells, and HMGB3 depletion reduces cisplatin adduct removal from cisplatin-resistant A2780/CP70 human ovarian cancer cells. (**A**) Schematic representation showing the N-terminal GFP-HMGB3 fusion protein construct. (**B**) A2780 cells expressing GFP-tagged HMGB3 show foci formation after 2 μM cisplatin treatment at 2 h and 24 h. (**C**) Schematic outline of foci formation and slot blot experimental design. (**D**) Representative western blot showing HMGB3 knockdown (~80% depletion) in human A2780 cells and A2780/CP70 cells. (**E**) Cisplatin–DNA adduct removal in cisplatin-resistant A2780/CP70 cells as a function of HMGB3 depletion, as assessed by slot blot assays using an anti-cisplatin DNA antibody (CP9/19 from Abcam) (top panel) with mouse secondary antibody and visualized with ECL and associated quantification of the total band intensity (average of three experiments). The *p*-value was calculated using a *t*-test, and values less than 0.05 were considered significant. ** indicates values less than 0.005. Methylene blue staining showing loading of genomic DNA and effective immobilization of the DNA onto the nitrocellulose membrane (bottom panel). (**F**) Quantification of slot blot band intensity showing remaining cisplatin adducts in the HMGB3-depleted samples from three independent experiments (N = 3); error bars = ±SD. (**G**) Quantification of the fold change in band intensity after 24 h and 120 h compared to the 0 h samples, suggesting a persistence in cisplatin adducts as a function of HMGB3 depletion in the A2780/CP70 cells. **, *p* < 0.01.

Our previously published studies showed that HMGB3 depletion sensitized cisplatin-resistant A2780/CP70 human ovarian cancer cells to cisplatin and induced apoptosis [30]. Therefore, the role of HMGB3 in cisplatin-induced DNA damage repair was investigated in these ovarian cancer cells [30]. A GFP-tagged HMGB3 construct was developed (Figure 4A), and expression in human A2780 cells was confirmed (Supplementary Figure S4). GFP-HMGB3 foci formation was observed at 2 h and 24 h following cisplatin treatment (at 2 µM), with the 24-h time point exhibiting an increased number of foci compared to the 2-h time point (Figure 4B). These results suggest that HMGB3 was recruited to cisplatin-induced DNA damage in A2780 ovarian cancer cells.

One mechanism of cisplatin resistance in ovarian cancer cells is more efficient removal of cisplatin–DNA adducts, thereby reducing drug efficacy [5]. Thus, we determined whether HMGB3 is associated with cisplatin adduct removal in the ovarian cancer cells. To measure this, slot blot assays were conducted in human cisplatin-sensitive A2780 and cisplatin-resistant A2780/CP70 cells (with or without HMGB3 depletion) following treatment with 2 µM cisplatin and collected for cisplatin adduct analysis at 1-, 24-, and 120-h post-treatment (Figure 4C,D). In A2780 cisplatin-sensitive cells, cisplatin adducts increased ~3-fold (consistent with the foci formation results) from 1 to 24 h and decreased ~4.6-fold from 24 h to 120 h but were still present at 120 h (Figure 4E–G). In contrast, in A2780/CP70 cells, cisplatin removal was nearly complete by 120 h. However, when HMGB3 was depleted from the resistant A2780/CP70 cells, there was a substantial accumulation of cisplatin adducts at 120 h with an ~3-fold increase from the 1-h timepoint, resulting in a significant (*p* < 0.005) difference in the number of lesions remaining as compared to the cells without HMGB3 depletion. These findings are consistent with a role for HMGB3 in removing cisplatin lesions in ovarian cancer cells. Moreover, at 120 h, both the A2780 and A2780/CP70 cells show a negative fold change in cisplatin lesions over time, while the HMGB3-depleted A2780/CP70 cells show a positive fold change in lesions over time, suggesting that HMGB3 may be facilitating efficient cisplatin–DNA adduct removal in A2780/CP70 cisplatin-resistant ovarian cancer cells.

### 2.5. HMGB3 Knockout Downregulates Several Cell Cycle-Associated and Chromatin Assembly-Associated Genes

**Figure 5:**
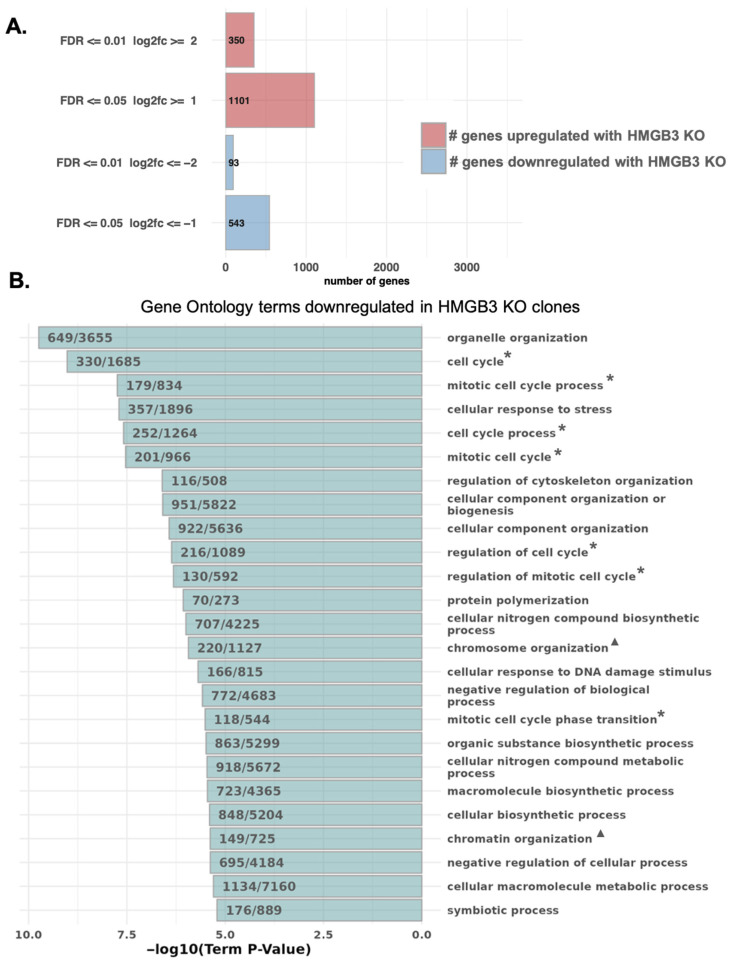
TagSeq reveals that HMGB3 knockout alters the expression of genes associated with the cell cycle and chromatin organization in human U2OS cells. (**A**) Bar graph depicting the number of differentially expressed genes between the three HMGB3 knockout clones and the wild-type cell line (red = upregulated in HMGB3 knockout clones, blue = downregulated in HMGB3 knockout clones). Genes that are upregulated and downregulated are further separated: first, into the number of genes with a false discover rate (FDR) less than or equal to 0.5 and a log2 fold change (log2fc) less than or equal to 1; and second, into those that have an FDR less than or equal to 0.01 and a log2fc less than or equal to 2. (**B**) Results from gene ontology (GO) analysis of biological processes impacted by HMGB3 knockout, showing that multiple chromatin and nucleosome assembly genes are significantly downregulated in the HMGB3 knockout clones compared to the wild-type cell line. Some of the terms of interest include those related to the cell cycle (*) and to chromatin organization (^▲^).

Previously, we found that HMGB3 facilitates transcription of the DNA damage response kinases, *ATR* and *CHK1*, in human ovarian cancer cells [30]. To determine the extent to which HMGB3 may alter transcription of other genes, we conducted a global TagSeq [43,44] experiment with three HMGB3 knockout human U2OS clones and WT U2OS cells. This assay was performed to investigate whether gene expression data could inform on the mechanisms in which HMGB3 may function and to provide future directions of research. The TagSeq results revealed differences in gene expression as a function of HMGB3. There were ~350 differentially expressed genes upregulated and 93 downregulated in all three HMGB3 knockout clones as compared to the WT cells, with a log2 fold change of ≥2 and a false discovery rate (FDR) of ≤0.01 (Figure 5A). There were an additional 750 genes upregulated and 450 genes downregulated in the HMGB3 knockout clones with a log2 fold change of ≥1 and FDR of ≤0.05. The top genes that were up- and downregulated can be found in Supplementary Tables S1 and S2, respectively. Of interest, gene ontology (GO) analysis of biological processes revealed that multiple cell cycle- and chromatin assembly-associated genes exhibited significantly lower expression in the HMGB3 KO clones than in the WT U2OS cells (Figure 5B). Some of the top 10 downregulated biological process terms related to the cell cycle were cell cycle (GO:0007049; *p* < 0.01), mitotic cell cycle process (GO1903047; *p* < 0.01), cell cycle process (GO:0022402; *p* < 0.01), mitotic cell cycle (GO:0000278; *p* < 0.01), and regulation of cell cycle (GO:0051726; *p* < 0.01). For chromatin assembly-associated terms, in the top 100 significantly downregulated biological process terms, chromosome organization (GO:0051276; *p* < 0.01), chromatin organization (GO:0006325; *p* < 0.01), chromatin assembly or disassembly (GO:0006333; *p* < 0.01), chromatin organization involved in regulation of transcription (GO:0034401; *p* < 0.01), chromatin assembly (GO:0031497; *p* < 0.01), and DNA packaging (GO:0006323; *p* < 0.01) were downregulated. These results suggest that HMGB3 may play a role in chromatin organization.

## 3. Discussion

HMGB3 is an architectural protein with the potential to influence DNA damage repair via binding to and bending DNA [18]. HMGB3 is similar in sequence and domain structure to its well-studied family member, HMGB1 [18], which is known to be involved in DNA repair, including roles in NER [7,8,9,10], mismatch repair (MMR), and base excision repair (BER) [27]. More specifically, in NER, HMGB1 facilitates error-free repair of intrastrand crosslinks and ICLs, binding to and bending the damaged DNA, further aiding in the recruitment of NER proteins, such as XPA and RPA, resulting in less mutagenic repair [8,9,10,29]. In MMR, HMGB1 has been found, in conjunction with MutL Protein Homolog 1 (MLH1), MutS Homolog 2 (MSH2), and Replication Protein A1 (RPA), to aid in damage recognition and to facilitate excision by exonuclease 1 (EXO-1) [27,45,46]. In BER, HMGB1 binds a BER intermediate and impacts apurinic/apyrimidinic endonuclease (APE1) and flap endonuclease 1 (FEN1) activity during lesion excision [27,47,48]. HMGB3 depletion can sensitize cancer cells to several DNA-damaging drugs [30,31,32,33,34], and given the physical similarities to HMGB1, it is feasible that these two proteins have somewhat redundant functions. However, our results suggest that HMGB3 is involved in DNA damage processing of DNA crosslinks in a fashion distinct from that of HMGB1.

**Figure 6:**
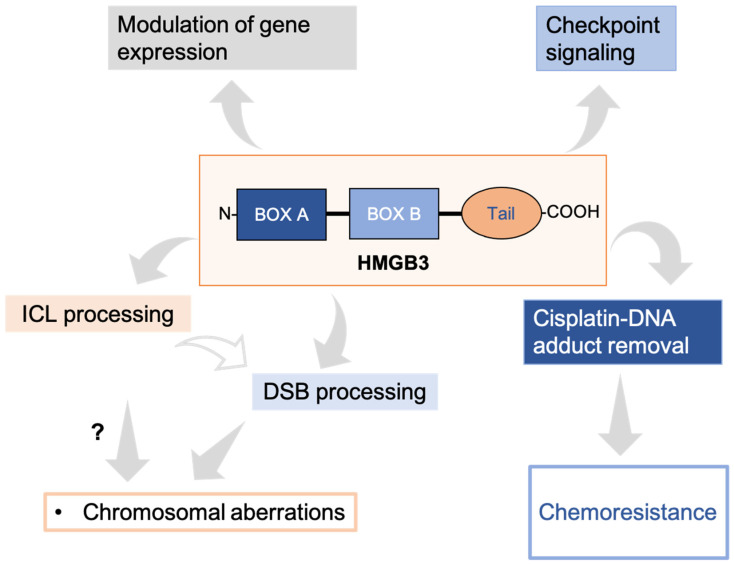
The emerging roles of HMGB3 in multiple cellular processes. HMGB3 is involved in the mutagenic processing of ICLs, likely through a DSB repair mechanism, and is associated with genomic stability. HMGB3 is also involved in cisplatin–DNA adduct processing in ovarian cancer cells. In addition, it is involved in checkpoint signaling following DNA damage in ovarian cancer cells. Further, expression of many genes was substantially modified by either up- or downregulation in HMGB3 knockout U2OS cells.

Here, we provide evidence for a role of HMGB3 in DNA damage processing and uncover new evidence for how HMGB3 may be contributing to cisplatin resistance in ovarian cancer cells. Interestingly, our results suggest that HMGB3 plays only a minor role in NER, which is in contrast to the substantial evidence for its family member, HMGB1, being an NER cofactor [7,8,9,10]. Instead, we found that HMGB3 is involved in ICL and DSB repair, and that in response to cisplatin treatment, HMGB3 forms foci and aids in cisplatin adduct removal. Lastly, gene expression analysis indicates that the expression of a variety of genes is altered by the presence (or absence) of HMGB3, including those involved in the cell cycle and chromatin organization, among others. Together, these data suggest that HMGB3 has multiple roles in the cell related to DNA damage repair, as outlined in Figure 6.

We first investigated a potential role for HMGB3 in NER, given the structural similarity between HMGB3 and HMGB1 [18]. Previously, we found that HMGB1 knockout in mouse embryonic fibroblasts (MEFs) has a profound effect on decreasing survival in response to UV damage [8]. Here, HMGB3 knockout in human U2OS cells does not appear to have a large effect or a significant effect at higher doses of UV irradiation. Additionally, we have previously found that HMGB1 depletion in mouse cells delays the removal of both CPDs and 6-4 PP following UV irradiation [8], while here, HMGB3 knockout in human U2OS cells does not appear to have an effect on CPD removal and may in fact facilitate 6-4PP removal.

Moreover, mutagenesis assays revealed that with HMGB1 knockout in MEFs, the mutation frequencies increased in response to UV damage [8], while here, in HMGB3 knockout human U2OS cells, the mutation frequencies were not significantly different than those in the WT U2OS cells, indicating there is not a substantial mutagenic impact of HMGB3 on UV lesion processing. Overall, in contrast to HMGB1, these results suggest that HMGB3 does not play a significant role in NER.

A potential role for HMGB3 in ICL repair was investigated as well. HMGB1 has been shown to be involved in ICL repair, albeit through NER, and HMGB3 has also been implicated in ICL repair in the case of site-directed psoralen-induced ICLs [30]. Additionally, HMGB3 confers resistance to cisplatin [30], a drug in which ICLs contribute to its toxicity [49], also indicating a potential role for HMGB3 in ICL repair but in an NER-independent mechanism. Evidence supporting a role of HMGB3 in ICL repair includes an increase in chromosomal aberrations and an increase in mutation frequencies following ICL treatment in HMGB3-depleted human cells compared to WT cells. Further, HMGB3 depletion in human cells results in an increase in the number of ICL-induced large deletions (from 0% to >30%) of the mutants characterized. NER processing does not typically result in large deletions, though processing by DSB repair pathways involved in ICL repair could lead to this outcome. Conversely, our previously published studies of HMGB1 depletion on ICL-processing primarily resulted in point mutations [10]. This indicates that HMGB1 and HMGB3 differ with respect to the mutagenic outcome resulting from ICL processing, suggesting that they are involved in different damage processing pathways.

Since HMGB3 appears to be involved in ICL processing, likely not via NER, but rather through DSB repair pathways, we investigated the potential role(s) of HMGB3 in several DSB repair pathways. First, we found an increased sensitivity to zeocin (a DSB-inducing agent) in HMGB3-depleted U2OS cells compared to WT U2OS cells. Further, we observed that with HMGB3 depletion, SSA is less efficient in processing ICLs. In addition, HMGB3 depletion resulted in a decrease in the frequency of HDR, generally considered an error-free DSB repair process. If there is a decrease in HDR following HMGB3 depletion, then an increase in the number of large deletions as seen from mutation screening could result if a more error-generating pathway, such as SSA, was used as an alternative pathway to process DSBs, causing a pathway shift to occur [38,50]. Since a decrease in SSA was also observed, this may not be the exact mechanism. However, the two assays used here for HR and SSA are not directly comparable, and the increase in large deletions from ICL processing observed with HMGB3 knockdown could still be due to a shift in DSB repair processing following HMGB3 depletion, where SSA, which is still occurring, is responsible for the mutations that are observed. NHEJ does not appear to be affected by HMGB3 depletion, indicating that end resection could be an important determining factor for HMGB3 in DSB repair pathways. End resection can be affected by the cell cycle via cyclin-dependent kinase (CDK)-mediated phosphorylation of various substrates [38,50], a process highly correlated to the cell cycle, which gene expression analysis revealed is affected in HMGB3-depleted cells. Nonetheless, depletion of HMGB3 in the context of ICL processing in human cells appears to contribute to genomic instability.

The role of HMGB3 in response to treatment with the chemotherapeutic drug, cisplatin, was further investigated by observing foci formation as a function of time and subsequently removal of cisplatin-adduct containing DNA via slot blot assays. Foci formation of HMGB3 in response to cisplatin treatment indicates a response in the nucleus of HMGB3 to DNA damage. The slot blot data support this, indicating that cisplatin-specific DNA damage removal is significantly delayed in HMGB3-depleted chemoresistant A2780/CP70 ovarian cancer cells. These data are consistent with our previously published results, supporting a role for HMGB3 in cisplatin resistance [30], which may in part be attributable to its role in cisplatin–DNA adduct processing. In conjunction with the evidence that HMGB3 is involved in ICL processing via DSB repair pathways, but not NER of intrastrand crosslinks, the potential direct influence of HMGB3 on cisplatin–induced DNA damage repair processing could primarily be due to cisplatin-induced ICLs rather than cisplatin-induced monoadducts.

As mentioned above, differences in the expression of groups of genes were observed in the GO analysis of biological processes between the WT and HMGB3 knockout cells. In addition to an overall downregulation of cell cycle-associated genes, there are also indications from the GO analysis that genes associated with chromatin organization could be impacted by HMGB3, consistent with HMGB3 being an architectural protein, and consistent with the relaxed chromatin appearance in HMGB3-depleted cells, as seen in the metaphase spreads. The result that chromatin organization genes are impacted as a function of HMGB3 could also indicate that HMGB3 may be influencing DNA damage repair in a more indirect manner via transcriptional regulation. In fact, HMGB1 and HMGB2 have previously been described to act in conjunction with transcription factors, including *p53* [51,52], and we have also found that HMGB3 is involved in facilitating transcription of the DNA damage response kinases, *ATR* and *CHK1*, in human ovarian cancer cells [30]. HMGB3 is also known to be associated with the Wnt/β-catenin signaling pathway in both hematopoietic stem cells [23] and in cancer cells, including in epithelial ovarian cancer [53], cervical cancer [54,55], and colorectal cancer [34,56,57]. Therefore, another potential contributing factor could be that HMGB3 knockdown has indirect effects on cell cycle processes via the Wnt/β-catenin signaling pathway. Future studies could reveal the extent to which HMGB3 impacts chromatin and transcriptional regulation, either directly or indirectly, and whether this impact has an important effect on its role(s) in chemotherapeutic DNA damage repair.

In conclusion, HMGB3 appears to be involved in ICL and DSB processing in human cells, a novel finding for this protein, in a fashion that is distinct from that of HMGB1, as the role for HMGB3 in NER appears to be minimal. This is a notable result, considering the structural and sequence similarities between the two proteins. Moreover, additional insight was gained into the potential mechanism(s) for how HMGB3 impacts cisplatin resistance by possibly facilitating more efficient DNA adduct removal, and into the effects of HMGB3 on the modulation of gene expression. Future investigation on how HMGB3 is involved in ICL and DSB repair, and subsequently, drug resistance in cancer cells, may allow for the development of improved therapies for cancer treatment and to improve the quality of life of cancer patients.

## 4. Materials and Methods

Cell Culture and siRNA Transfection: The human U2OS knockout clones, and corresponding wild-type (WT) cells, were grown in McCoy’s 5A media (Iwakata and Grace modified) with L. Glutamine, 10% FBS, and 1% Pen-Strep (Cat #10-050-CV, Corning Inc., Corning, NY, USA), according to ATCC guidelines, in a 37 °C incubator (5% CO_2_). All other U2OS cells, human ovarian cancer cells (A2780), and cisplatin-resistant cells (A2780/CP70) were cultured as previously described [10,30]. The cisplatin resistance was maintained in the cisplatin-resistant ovarian cancer cells (A2780/CP70) as previously described [30]. All siRNA-mediated transfections of HMGB3 siRNA and control (non-targeting/mock) siRNA were carried out as previously described [10,30].

Isolation of HMGB3 KO Clones: Human U2OS cells were obtained (Synthego, Redwood City, CA, USA), pre-treated with CRISPR-Cas9 targeting of *HMGB3*, or left untreated. Individual cells were sorted into 96-well plates with a flow cytometer using the LIVE/DEAD Fixable Green Dead Cell Stain Kit (Cat L34969, Invitrogen, Waltham, MA, USA). DNA was isolated from individual clones and sequenced at the University of Texas at Austin DNA Sequencing Facility using Synthego-designed primers (F: 5′-TGCTCTGAGAGGTGGAAGGT-3′; R: 5′-TCCTTCTTCTTCTTGCCTCCC-3′), which encompass the CRISPR-Cas9 gRNA sequence, 5′-GUUCUUUGCAGACGAUGUCC-3′, that was used by Synthego to knock out *HMGB3*. Clones that were identified as potential knockout clones were then tested for HMGB3 protein levels via Western blot. Briefly, protein concentration was measured using the Pierce BCA Protein Assay Kit (Cat #23225, ThermoFisher Scientific, Waltham, MA, USA), and equal amounts of protein were subjected to electrophoresis on 4–15% gradient Western blot gels and transferred to a nitrocellulose membrane. The membrane was blocked in 5% milk in TBST, and primary antibody incubation was conducted overnight in 5% milk in TBST with primary antibodies rabbit α-HMGB3 (Cat: ab75782, abcam, Cambridge, UK) and rabbit α-beta-Actin (Cat: ab8227, abcam) at a 1:1000 dilution at 4 °C. Secondary antibody incubation was conducted for two hours at room temperature in 5% milk in TBST with goat α-mouse (Cat #170-6515, BioRad, Hercules, CA, USA). Imaging was conducted using the Clarity Western ECL Kit (Cat: #1705061, BioRad) and the BioRad GelDoc imaging system. Three HMGB3 KO clones were identified and used in subsequent experiments.

Clonogenic Assays: Cells were seeded in 60 mm cell culture dishes, left in the 37 °C incubator overnight to adhere, and irradiated the following day with either UVC (254 nm) at a dose of 3 J/m^2^, 6 J/m^2^, 10 J/m^2^, or Zeocin at a dose of 15 J/m^2^, or with 6.25 mg/mL, 25 mg/mL, 50 mg/mL, 100 mg/mL, or 200 mg/mL. Fresh 37 °C media was added, plates containing the undamaged control cells were left untreated but were washed with PBS, and fresh 37 °C media was added. Following a 24-h incubation at 37 °C, 1000 cells were seeded on three fresh 60 mm cell culture dishes and left to incubate at 37 °C until colonies were large enough to visualize by staining. Cells were fixed to the cell culture dish with 95% EtOH and then stained with 0.125% crystal violet for quantification. Four replicates were collected. Statistical analyses were performed with GraphPad Prism software (version 9.5.1) using a two-way ANOVA (Dunnett’s multiple comparisons test), *p* < 0.05.

Cell Line Construction and Induction of DNA Double-Strand Breaks: A NHEJ reporter construct (IRES-TK-EGFPKV) was built in-house based on the IRES-TK-EGFP construct reported by [42]. Briefly, the GFP sequence from the pmaxGFP plasmid (Lonza, Basel, Switzerland) was cloned into the pIRESpuro3 plasmid (Clontech, San Jose, CA, USA). Subsequently, the Herpes simplex virus-thymidine kinase (HSV-TK) gene, including the promoter through the stop codon, was amplified with primers containing inverted I-SceI target sequences and cloned 5′ to the GFP sequence. Also contained in the HSV-TK-3′ primer were stop codons in all three reading frames to ensure GFP was not expressed. The IRES-TK-EGFPKV reporter construct was introduced into U2OS cells using Xfect transfection reagent (Clontech, San Jose, CA, USA), and stably integrated clones were selected for in 1.5 μg/mL puromycin (Sigma-Aldrich, St. Louis, MO, USA).

For an HR reporting system, we obtained U2OS cells containing the DR-GFP reporter substrate [40] from the laboratory of Dr. Kyle Miller. We next integrated an inducible system to temporally control the formation of I-SceI DSBs, so that DSBs could be formed at maximum siRNA-mediated protein depletion. To do this, the mCherry-ISceI-GR plasmid [58] was obtained from the laboratory of Dr. Michal Goldberg and introduced into U2OS cells containing the IRES-TK-EGFPKV or DR-GFP reporter substrates using Xfect transfection reagent (Clontech). Stably integrated cells were selected for in 400 μg/mL G418 (Invivogen, San Diego, CA, USA) and then sorted for low mCherry and no GFP expression using a FACS Aria II Cell Sorter. After sorting, an individual clone was selected from each group that displayed a low background of GFP-expressing cells (<0.5%) and was highly inducible by exposure to dexamethasone. U2OS cells with the NHEJ or DR-GFP reporter construct were grown in Optimem media (Life Technologies, Waltham, MA, USA) with 10% charcoal stripped serum. To deplete HMGB proteins, 400,000 cells were transfected with HMGB3 siRNA smart pool (Thermo Scientific) using Lipofectamine RNAiMAX transfection reagent (Invitrogen) according to the manufacturer’s protocol and plated onto a 60 mm petri dish to achieve a 50% confluence in 24 h. After 24 h, the cells were transfected a second time with HMGB3 siRNAs. Twenty-four hours following the second transfection, the cells were treated with 10^−7^ M dexamethasone (Sigma) to translocate I-SceI into the nucleus and induce DSBs. Forty-eight hours later, the cells were collected for FACS analysis.

Slot Blot Assays: Cells were seeded in 60 mm petri dishes and left overnight to adhere at 37 °C. The plated cells were then treated either with UVC at a dose of 3 J/m^2^ or at 6 J/m^2^, or with 2 μM cisplatin (as previously described [30]), and the control plates were left untreated. Fresh 37 °C media was added to the plates, and cells were then collected at either 0-, 0.5-, 1-, 2-, 4-, 6-, and 24-h post-UVC treatment or at 1-, 24-, and 120-h post-cisplatin treatment. Cell pellets were thawed, resuspended in 200 μL of PBS, and 4 μL Qiagen RNase A (100 mg/mL) was added to each sample. Then, the samples were processed using the QIAamp DNA Mini Kit (Cat #51106) following the manufacturer’s protocol, and the protocol utilized in Park and Kang (2015) [59]. DNA concentrations were measured on a Nanodrop (ThermoFisher). For samples that were probed for cyclobutene pyrimidine dimers (CPDs), 100 ng of DNA was used, and for the samples that were probed for 6-4 photoproducts (6-4 PPs), 2 μg of DNA was used, and the volumes were brought up to 200 μL with either 0.4 M NaOH and 10 mM EDTA or autoclaved dH_2_O, respectively. For samples probed for cisplatin–DNA adduct damage, 750 ng of genomic DNA was used. Samples were heated on a heat block at 95 °C for 10 min and immediately placed on ice. To the samples that were probed for CPDs and cisplatin adducts, an equal volume of 4 °C ammonium acetate was added, and to the samples that were probed for 6-4 PPs, an equal volume of 4 °C dH_2_O was added. The slot blot apparatus (Bio-Dot SF Microfiltration, BIO RAD) was prepared as per the manufacturer’s instructions with a nitrocellulose membrane. 1X TE was vacuumed through the apparatus, samples were then loaded and vacuumed, followed by 2X saline-sodium citrate buffer. The blot was air-dried at 80 °C in a hybridization oven for 2 h. Blots were rehydrated in TBST and blocked in 5% milk in TBST. Blots were incubated with primary antibody in 5% milk in TBST overnight at 4 °C. Separate blots were used for 6-4 PPs (mouse anti-6-4PP, Cat: CAC-NM-DND-002, Cosmo Bio USA, Carlsbad, CA, USA) and CPDs (mouse anti-thymine dimer, Cat: MC-062, Kamiya, Tukwila, WA, USA) for each experiment in the UVC assay. Cisplatin adducts were detected with the anti-cisplatin DNA adduct (C9/19 antibody, abcam, Cambridge, UK) primary antibody. Secondary antibody incubation was conducted for two hours at room temperature in 5% milk in TBST with goat α-mouse, Cat #170-6516, BioRad for the 6-4 PP, CPD blots, and cisplatin blots. Blots were imaged using the Clarity Western ECL Kit (Cat: #1705061, BioRad) and BioRad GelDoc imaging system. Blots were then probed with SYBR gold at a 1:10,000 ratio in TBST, and imaged upside-down on the BioRad GelDoc imaging system to obtain a clear image. Statistical analyses were performed via GraphPad Prism software using a two-way ANOVA (Dunnett’s multiple comparisons test), *p* < 0.05.

Mutagenesis Assays: Cells were either seeded in 60 mm petri dishes and left overnight at 37 °C to adhere for the HMGB3 knockout cells or a reverse transfection was performed while plating, followed the next day by a forward transfection for the samples undergoing HMGB3 knockdown (as previously described [10]). A mutation-reporter plasmid was damaged, where either plasmid psp189 was irradiated with UVC (350 J/m^2^) or plasmid pSupFG1was treated with a pTFO-ICL, as previously described [10]. Two μg of treated plasmid or control (untreated) plasmid were then transfected into the respective cells using GenePORTER (Cat: T201075, Genlantis, San Diego, CA, USA). Cells were collected approximately 48 h after transfection. Pellets were resuspended in 100 μL Qiagen buffer P1 (Cat: #19051), lysed with 100 μL Qiagen buffer P2 (Cat: #19052), and neutralized with 100 μL buffer N3 (Cat: #19064). Then the samples were subjected to phenol/chloroform extraction and EtOH precipitation, followed by *Dpn1* digestion to remove plasmids not replicated in the U2OS cells. This was followed by a second phenol/chloroform extraction and EtOH precipitation. DNA pellets were resuspended in 12 μL Qiagen EB buffer (Cat: #19086). The collected DNA was used to transform into MBM7070 *E. coli* cells (Lucigen, Middleton, WI, USA) for blue-white mutation screening. The number of mutants and total colonies were used to determine the mutation frequency (number of mutant colonies per 10,000 colonies). At least 10,000 colonies were counted for each treatment. Statistical analyses were performed via GraphPad Prism software using a one-way ANOVA, *p* < 0.05. Mutant colonies were sent for Sanger sequencing at the University of Texas at Austin DNA Sequencing Core Facility and compared to the parent plasmid sequence. The number of types of mutations was recorded to determine the mutation spectra.

*TagSeq Sample Preparation*: The three identified HMGB3 KO clones, along with the WT U2OS cells, were used for the TagSeq experiments. Cells of each type were maintained in three separate flasks prior to collection for replicate samples. RNA extraction was conducted using the Qiagen RNeasy micro kit (Cat: #74004) following the manufacturer’s protocol, with the exception of completing all of the centrifugation steps at 4 °C. RNA concentrations were then measured using a Nanodrop, flash-frozen, and stored at −80 °C. Four replicates were collected. The samples were sequenced using the TagSeq method [43,44] by the University of Texas at Austin Genomic Sequencing and Analysis Facility (GSAF).

### Gene Expression Analysis Methods

*Experimental Libraries*: Four cDNA libraries were constructed, each with four replicates: *WT*–wild-type human U2OS cells, *KO1*-CRISPR HMGB3 knockout clone #2, *KO2*-CRISPR HMGB3 knockout clone #5, *KO3*-CRISPR HMGB3 knockout clone #6.

TagSeq Library Preparation: Library preparation and sequencing for TagSeq [43,44], a form of 3′ RNA sequencing, were performed by the GSAF at the University of Texas at Austin. Total RNA was isolated from each sample (0.375 mL) by addition of Trizol (1.125 mL; Thermo Fisher), and the sample (1.4 mL) was transferred to a Phasemaker tube (Thermo Fisher). Total RNA was extracted following the protocol supplied by the manufacturer and further cleaned up using an RNeasy MinElute Cleanup Kit (QIAGEN, Germantown, MD, USA). RNA integrity number (RIN) was measured using an Agilent Bioanalyzer, and 100 ng of RNA was used for the TagSeq protocol. The fragmentation/RT mix was prepared, added to each RNA sample, heated to 95 °C for 2.5 min on a Thermocyler, then immediately put on ice for 2 min. After cooling and the addition of the template switching oligo and SmartScribe RT, the fragmented RNA reaction was incubated at 42 °C for 1 h and at 65 °C for 15 min. Next, an AmPure bead clean-up was completed for the cDNA before it was amplified for 18 cycles, also incorporating the Illumina sequencing primer site, followed by another cleanup. The remaining portions of the Illumina adapter (the i5 and i7 indices) were then incorporated through an additional 4 cycles of PCR. Final libraries were quantified with PicoGreen, then pooled equally for size selection using the Blue Pippin from 355–550 bp. The resulting libraries were sequenced using an Illumina Novaseq instrument (100-nucleotide single reads).

Sequence Data Pre-processing: FASTQ datasets were post-processed [60] (TagSeq post-processing based on tag-based_RNAseq code as of August 2019) in order to collapse duplicates based on molecular indices. Sequencing data quality, both before and after TagSeq pre-processing, was evaluated using the Babraham Bioinformatics FasQC tool [61] (v0.11.9), and reports were aggregated with the MultiQC [62] program (v1.9).

Alignment: Single-end pseudo-alignment was performed against the human transcriptome (GENCODE [63] v42 transcript sequences) using kallisto [64] (v0.45.0) with options -l 200 -s 50 --single-overhang --bias. Kallisto’s estimated counts were used for differential expression analysis.

Differential Gene Expression Analysis: Downstream analysis of transcript abundance data was performed in R [65] (v4.0.3) following protocols outlined in Bioconductor [66]. The tximport [67] package (v1.6.0) was first used to roll up transcript-level counts into gene-level counts provided to the DESeq2 [68] package (v1.18.1). Before further analysis, count data matrices were filtered to remove genes with fewer than 1 read across all included samples. Three contrasts were analyzed to explore the relationship between each of the knockout clones and the WT (e.g., KO1 vs. WT). A fourth model was analyzed, comparing all knockout samples to the WT. Differentially expressed genes (DEGs) reported are those with a maximum adjusted *p*-value of 0.05 and a log2 fold change greater than 1.0 or less than −1.0.

Gene Ontology (GO) Analysis: Gene ontology (GO) analyses were performed using R packages topGO [69,70] (ver. 2.42.0) and GO-MWU [71,72]. For topGO analysis (with org.Hs.eg.db 3.12.0), genes with a DESeq2 maximum adjusted *p*-value of 0.05 that were upregulated (log2 fold change ≥ 0.05) or downregulated (log2 fold change ≤ −0.05) were provided to topGO. The background gene universe consisted of observed genes that were assigned an adjusted *p*-value by DESeq2. topGO’s classic algorithm and Fisher’s exact test were used in count data mode. For GO-MWU, related GO terms are first combined into larger term category groups, then the significance of each GO category is determined by applying the Mann-Whitney U (MWU) test on the distribution of observed scores in each term group. Scores used for GO-MWU analysis were DESeq2’s log2 fold change and its z-like stat score. Scores for all genes that were assigned an adjusted *p*-value by DESeq were provided to GO-MWU, and only scores for non-redundant genes in each term category were used to model the score distribution. The individual MWU *p*-values were then subjected to multiple correction testing to obtain a false discovery rate (FDR). Ontology and human gene annotation databases [73] from release 1 January 2023 were used.

Metaphase Spreads: To visualize the chromosomal aberrations induced by ICLs, we performed metaphase spreads on cells treated with 10^−9^ M psoralen followed by 1.8 J/m^2^ UVA (365 nm) treatment in phenol red-free and serum-free media. Immediately after the treatment, the media was replaced with serum-containing media for recovery. Untreated cells were used as a control. 30 μL of the reagent 10 μg/mL KaryoMAX Colcemid (Thermo Fisher) was added to each plate to arrest the cells in metaphase for further cytogenic studies. Halting the cells in metaphase allows for the chromosomes to be in an X formation, which enables the clearest viewing of chromosomal aberrations. After incubating with Colcemid at 37 °C, 5% CO_2_ for 2 h, the cells were collected by trypsinization from the HMGB-depleted plates and placed in a 15 mL conical tube. They were then centrifuged for 5 min at 1000 rpm at room temperature. Once the supernatant was discarded, the cells were treated with 5 mL of warm hypotonic solution (0.075 M KCl) slowly and mixed well by flicking the tube to prepare a homogenous mixture where all the cells were exposed to the solution. The hypotonic solution allows for swelling of the cells and facilitates cell lysis. This tube was slowly rocked back and forth many times to ensure equal exposure of the cells, and then was incubated for 30 min at 37 °C, 5% CO_2_. Next, 100 μL of freshly prepared Carnoy’s fixative (3:1 ratio of methanol and glacial acetic acid, respectively) was added to the tube to inhibit the hypotonic solution, mixed well, and incubated at room temperature for 10 min. Subsequently, the cells were centrifuged at 24 °C at 1000 rpm for 5 min, and the supernatant was aspirated. Then, 1 mL of the fixative was slowly added to the tube, mixed well by flicking, and then the volume was brought up to 5 mL. The tube was then rocked several times, incubated for 10 min, and centrifuged again. This process was repeated twice more to complete the fixation of cells. The cells were then either stored in fixative at 4 °C for later use or were dropped onto slides immediately after. The slides (Fisherbrand frosted slides, precleaned) were twice washed with 2 mL of fresh fixative and allowed to air dry, and 100 μL of the cells suspended in fixative were then dropped using a pipettor from roughly 2.5 feet above the slide to facilitate lysis of the cells upon impact. The slides were then quickly blown upon to further facilitate lysis and subsequently placed in a 50 °C water bath for 30 s to help fix the lysed cells on the slides. The slides were then allowed to sit at room temperature overnight and stained with a 10% Giemsa solution after 24 h.

Single-strand Annealing Mutation-Reporter Assays: To determine the effect of HMGB protein depletion in U2OS, XPA- and XPF-deficient cells, 2 µg of plasmids containing TFO-directed ICLs or control plasmids were transfected using GenePORTER (Invitrogen) transfection reagent following the second round of HMGB1 or control siRNA transfections as per the manufacturer’s suggestions. Mutagenesis assays were performed as described (Christensen et al., 2004, Mol. Carcinog. [37]). In brief, cells were harvested 48 h post-transfection, and plasmid DNA was isolated using the QIAprep Spin Miniprep kit (QIAGEN Inc.). These plasmids were treated with *DpnI* restriction endonuclease for 1 h at 37 °C, followed by phenol/chloroform/isoamyl alcohol extraction and ethanol precipitation with 0.3 M sodium acetate (pH 5.2), and were re-suspended in 10 µL nuclease-free water. Mutations in the *supF* gene or correction of mutations in the *supF* gene due to processing of the TFO-directed ICLs were detected by transforming electro-competent *E. coli* cells MBM7070 (Lucigen) with 1 µL (~100 ng) of plasmid DNA and plated onto X-gal, IPTG, and ampicillin-containing agar plates for blue/white screening. A functional *supF* gene produces a blue colony, while a mutation in the *supF* gene produces a white colony. Mutation frequencies were measured by counting the blue colonies over the total number of colonies counted for the SSA assays. Three individual experiments were performed for each mutagenesis assay, and a total of 30,000 colonies were counted per experiment. The *p*-values were generated by Holm-Sidak one-way analysis of variance method. Mutation spectra were determined by sequencing the plasmids from white colonies using primers matching the ampicillin resistance gene and comparing them against the control sequences generated from the white colonies.

Homologous Recombination and NHEJ GFP Reporter Assays: A NHEJ reporter construct (IRES-TK-EGFPKV) was built in-house based on a previously reported construct, IRES-TK-EGFP [42]. Briefly, the GFP sequence from the pmaxGFP plasmid (Lonza) was cloned into the pIRESpuro3 plasmid (Clontech). Subsequently, the *Herpes simplex virus-thymidine kinase* (*HSV-TK*) gene, including the promoter through the stop codon, was amplified with primers containing inverted *I-SceI* target sequences and cloned 5′ to the GFP sequence. Also contained in the HSV-TK-3′ primer were stop codons in all three reading frames to ensure GFP was not expressed. The IRES-TK-EGFPKV reporter construct was introduced into U2OS cells using Xfect transfection reagent (Clontech), and stably integrated clones were selected for in 1.5 μg/mL puromycin (Sigma). For an HR reporting system, we obtained U2OS cells containing the DR-GFP reporter substrate [40] from the Kyle Miller laboratory. We next integrated an inducible system to temporally control the formation of *I-SceI* DSBs, so that DSBs could be formed at maximum siRNA-mediated protein depletion. To do this, the mCherry-ISceI-GR plasmid [58] was obtained from the Michal Goldberg laboratory and introduced into U2OS cells containing the IRES-TK-EGFPKV or DR-GFP reporter substrates using Xfect transfection reagent (Clontech). Stably integrated cells were selected for in 400 μg/mL G418 (Invivogen) and then sorted for low mCherry and no GFP expression using a FACS Aria II Cell Sorter (BD Biosciences, San Jose CA, USA). After sorting, an individual clone was selected from each group that displayed a low background of GFP-expressing cells (<0.5%) and was highly inducible by exposure to dexamethasone.

*Foci Formation Detection*: HMGB3_FWD 5′-TCACCATGGCTAAAGGTGACCCCAAG-3′ and HMGB3_REV 5′-CTTATTCATCCTCCTCCTCCTCCTC-3′ primers were used to clone the *HMGB3* gene from U2OS cDNA. N-terminal GFP-tagged HMGB3 was prepared via gateway cloning (Thermo Fisher) following the manufacturer’s suggested protocol. HMGB3_Fwd_N-GFP 5′-GGGG ACA AGT TTG TAC AAA AAA GCA GGC TTC ACC ATG GCT AAA GGT GAC CCC AAG-3′ and HMGB3_Rev_N-GFP 5′-GGG GAC CAC TTT GTA CAA GAA AGC TGG GTC TTA TTC ATC TTC CTC CTC TTC-3′ primers were used for gateway cloning. The N-GFP-HMGB3 product was sequenced to confirm the accuracy of the construct and was subsequently transfected into human A2780 cells using the Geneporter transfection reagent as mentioned above and selected with blasticidin (10 μg/mL) for 15 days. The cells were sorted, and single cells were plated for clonal selection. Clones were grown and checked for GFP-HMGB3 fusion protein expression by Western blotting. Clone 11 was used for subsequent foci formation experiments by plating 500 cells in glass-bottom culture dishes (Thermo Fisher). Cells were treated with 2 μM cisplatin and visualized with a Leica SP8 DLS confocal microscope at different time points, and data were collected.

## Figures and Tables

**Table 1:** Mutation spectra resulting from processing of a UVC-treated mutation reporter (psp189) in human U2OS cells as a function of HMGB3 knockout in three clones and wild-type U2OS cells.

Cell Line	Base Substitution	Insertion	Single Nucleotide Deletion	Large Deletion	Genomic Fusions	Total Sequenced
Wild Type	92%	-	-	8%	-	20
HMGB3 KO Cl.#2	92%	-	-	8%	-	10
HMGB3 KO Cl.#5	100%	-	-	-	-	10
HMGB3 KO Cl.#6	100%	-	-	-	-	10

**Table 2:** Mutation spectra resulting from TFO-directed ICL processing in human U2OS cells as a function of HMGB3 depletion.

Depletion of	Base Substitution	Insertion	Single Nucleotide Deletion	Large Deletion	Genomic Fusions	Total Sequenced
No depletion	100%	-	-	-	-	20
HMGB3	31.5%	21%	10.2%	36.3%	-	20

## Data Availability

The original data presented in the study are openly available in the NCBI Gene Expression Omnibus under accession number GSE285696.

## Supplementary Materials

The following supporting information can be downloaded at: https://www.mdpi.com/article/10.3390/genes16091044/s1, Supporting Materials and Methods [10,74]: Triplex-directed ICL formation; Supporting Materials and Methods: Electrophoretic mobility shift assays; Figure S1: Three HMGB3 knockout clones were isolated in human U2OS cells; Figure S2: Representative slot blot images of CPDs and 6-4PPs following UVC irradiation in wild type and HMGB3 knockout human U2OS cell lines; Figure S3: Sequencing results showing the mutation spectra on the mutation reporter with reference to the ICL position as a function of siRNA-mediated HMGB3 depletion in human U2OS cells; Figure S4: Assessment of GFP-tagged HMGB3 expression in human A2780 clones; Table S1: Top differentially expressed genes that are upregulated in HMGB3 knockout clones compared to wild type U2OS cells; Table S2: Top differentially expressed genes that are downregulated in HMGB3 knockout clones compared to wild type U2OS cells.

## Abbreviations

The following abbreviations are used in this manuscript:

HMGB3	High Mobility Group Box Protein 3
HMGB1	High Mobility Group Box Protein 1
NER	Nucleotide excision repair
ICL	DNA interstrand crosslink
pTFO-ICLs	Triplex-directed psoralen ICLs
DSB	Double-strand break
6-4 PPs	6-4 photoproducts
CPDs	Cyclobutene pyrimidine dimers
SSA	Single-strand annealing
HDR	Homology-directed repair
NHEJ	Non-homologous end joining
FDR	False discovery rate
GO	Gene Ontology
WT	Wild type
MMR	Mismatch repair
BER	Base excision repair
MLH1	MutL Protein Homolog 1
EXO-1	Exonuclease 1
APE1	Apurinic/apyrimidinic endonuclease
MEFs	Mouse embryonic fibroblasts
CDK	cyclin-dependent kinase
