# Supplementary material for: High-Mobility Group Box Protein 3 (HMGB3) Facilitates DNA Interstrand Crosslink Processing and Double-Strand Break Repair in Human Cells
Source: Genes (Basel). 2025 Sep 4;16(9):1044. doi: 10.3390/genes16091044 (PMC12470105; doi:10.3390/genes16091044)
Supplement: Supplementary file 1 [file genes-16-01044-s001.zip › genes-3848345-supplementary.pdf]

Genes

Supporting Information for

High-Mobility Group Box protein 3 (HMGB3) facilitates DNA interstrand crosslink processing and double-strand break repair in human cells

Jillian Dangerfield[1*], Anirban Mukherjee[2*], Wade Reh[3], Anna Battenhouse[4], Karen M. Vasquez[1^]

^Corresponding Author: Karen M. Vasquez
Email: karen.vasquez@austin.utexas.edu

This PDF file includes:

Supporting text
Figures S1 to S4
Tables S1 to S2
SI References

Supporting information text

Supporting materials and methods:

Triplex-directed ICL formation

Triplex-directed ICL containing DNA substrates were prepared as we have described[1]. Briefly, radio-labeled 57-bp duplexes (10^{-6} M) were incubated with psoralen-conjugated 30-mer TFOs (10^{-6} M) in a triplex binding buffer [10 mM Tris-HCl, pH 7.6, 10 mM $MgCl_2$, and 10% (vol/vol) glycerol] at 37 °C for 16 h. Samples were then irradiated with 1.8 J/cm^2 of UVA (365 nm) under a mylar filter to induce psoralen ICLs. The efficiency of triplex-ICL formation was assessed at ~90%.

Electrophoretic mobility shift assays

Electrophoretic mobility shift assays (EMSA) were performed as described[2]. Briefly, 10^{-9} M DNA substrates were incubated for 20 minutes at 30° C with 10^{-8} M and 10^{-9} M purified recombinant HMGB3 protein (Cat YSP5093, Speed Biosystems) in DNA-binding buffer (25 mM Tris-HCl pH 7.6, 100 mM NaCl, 1 mM DTT, 5 mM EDTA, 100 μg/mL BSA, 0.01% Nonidet P-40, and 10% glycerol) in a 10 μL reaction volume. The reactions were then resolved on 6% native polyacrylamide (acrylamide:bis acrylamide = 37.5:1) gels in 1× TBE (89 mM Tris-borate, pH 8.0, 2 mM EDTA) buffer for 2 hours at 4° C. Subsequently, gels were dried, exposed overnight, and visualized using a phosphorimager.

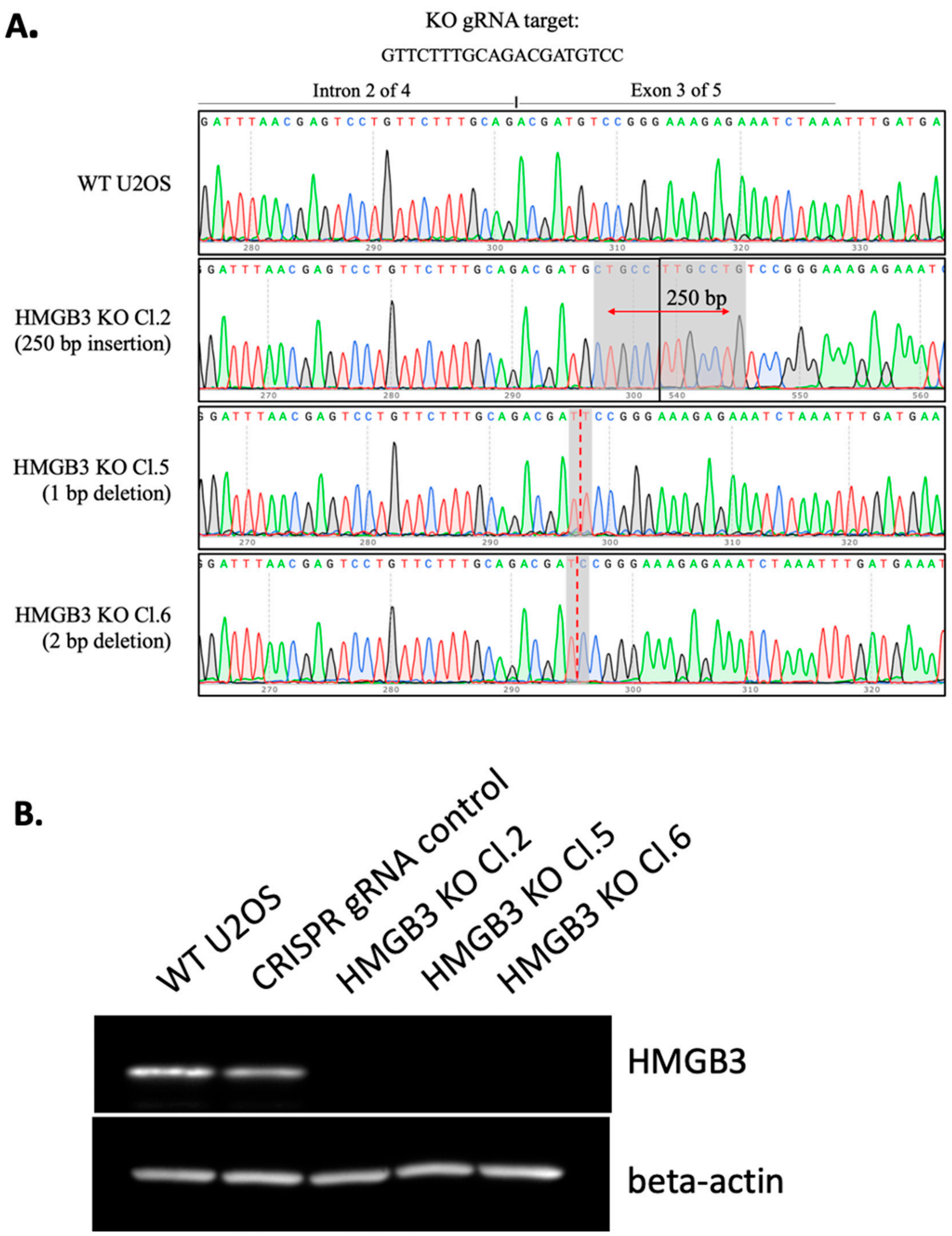

Figure S1: Three HMGB3 knockout clones were isolated in human U2OS cells. A. Sanger sequencing data at the site targeted by the CRISPR-Cas9 gRNA in box 1 of *HMGB3* in the U2OS wild type and three HMGB3 knockout clones. All of the mutations occurred in the 3rd exon of the gene as assessed by DNA sequencing. Clone 2 has a 250-base pair (bp) deletion, highlighted in gray, and clones 5 and 6 have a 1-bp and 2-bp deletion, respectively, the location of which is depicted by a dotted red line highlighted in gray. **B.** Western blot showing HMGB3 knockout in the three clones.

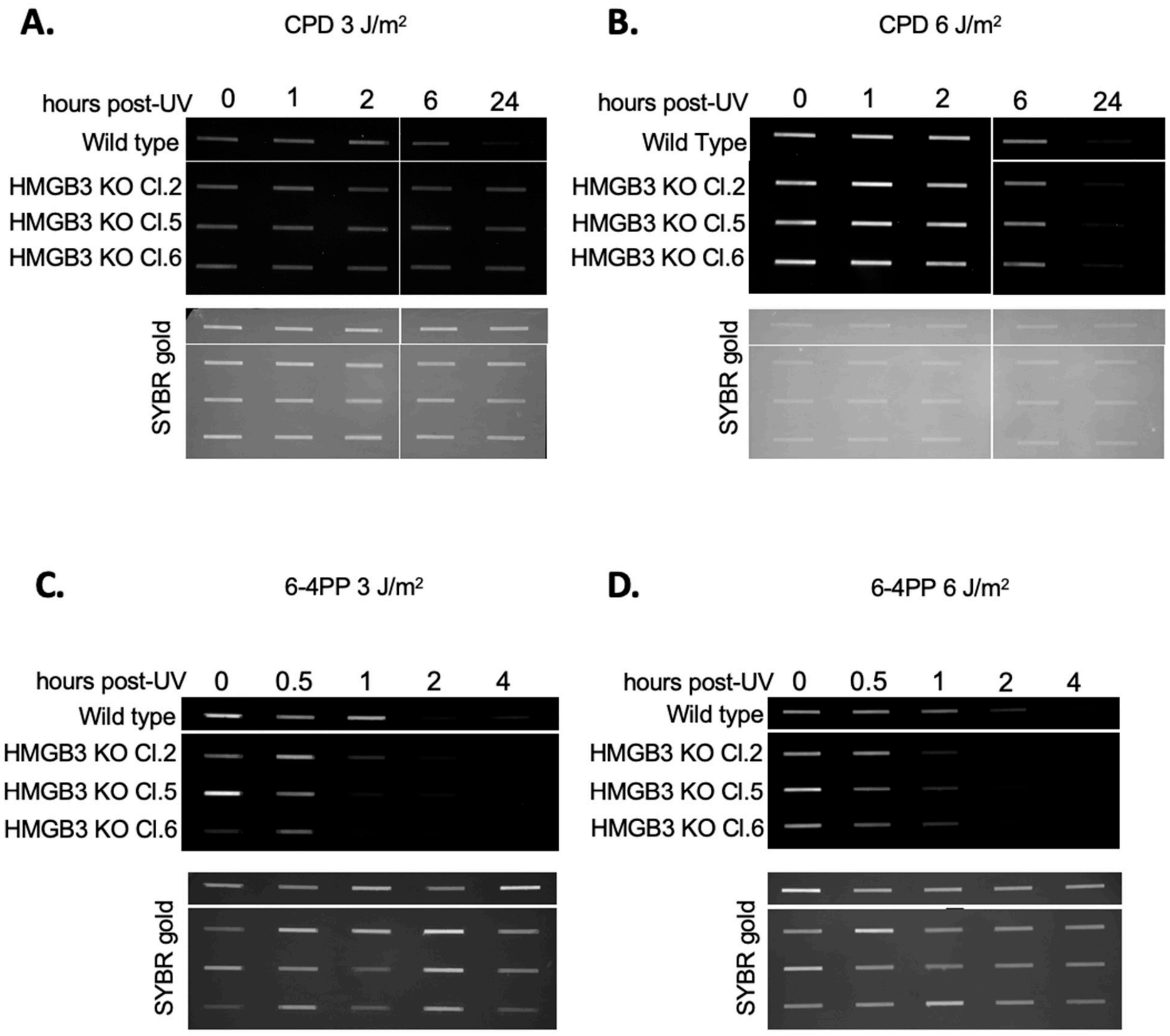

Figure S2: Representative slot blot images of CPDs and 6-4PPs following UVC irradiation in wild-type and HMGB3 knockout human U2OS cell lines. A. A representative image and quantification of a slot blot assay determining the effect of HMGB3 knockout on DNA damage removal following UVC treatment. Antibodies were used to detect 6-4 PPs (mouse anti-6-4PP, Cat: CAC-NM-DND-002, Cosmo Bio USA) or CPDs (mouse anti-thymine dimer, Cat: MC-062, Kamiya). There were no significant differences detected between the HMGB3 KO clones and the wild-type cell line in CPD removal following 3 J/m^2 at any time point, and following **B.** 6 J/m^2, there was a significant ($p<0.05$) increase in CPD removal at two hours post-UVC treatment in the HMGB3 KO clones compared to the wild-type cell line. There was a significant increase in 6-4 PP removal in the HMGB3 KO clones as compared to the wild-type cell line at both **C.** 3 J/m^2 at 0.5 hours and at one hour post-UVC treatment, and following **D.** 6 J/m^2 at 0.5 hours, one hour, and two hours post-UVC treatment; error bars = SEM.

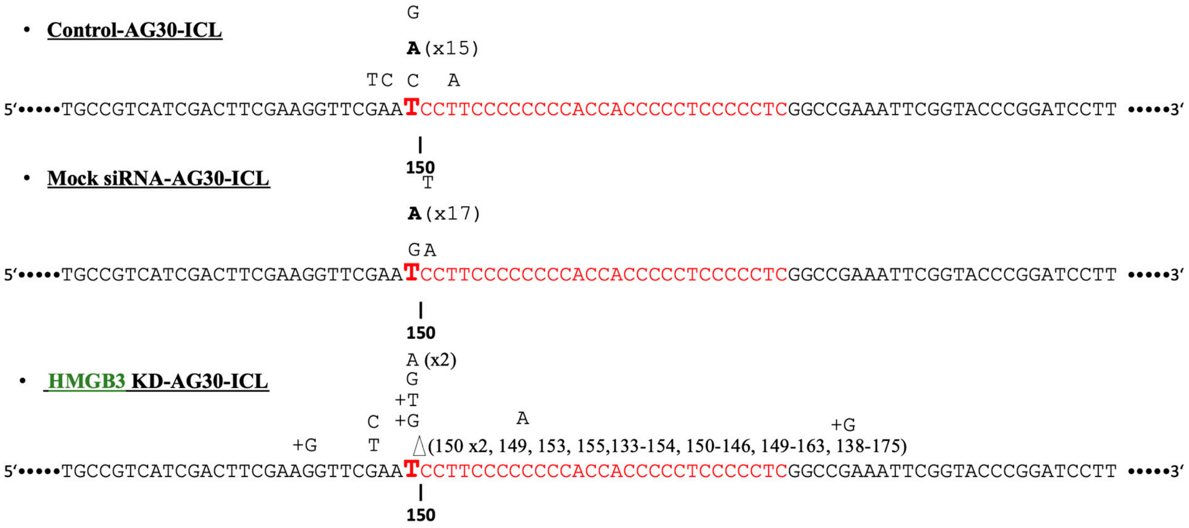

Figure S3: Sequencing results showing the mutation spectra on the mutation reporter with reference to the ICL position as a function of siRNA-mediated HMGB3 depletion in human U2OS cells. The highlighted T is the position of ICL, and all the changes are mentioned with respect to that position.

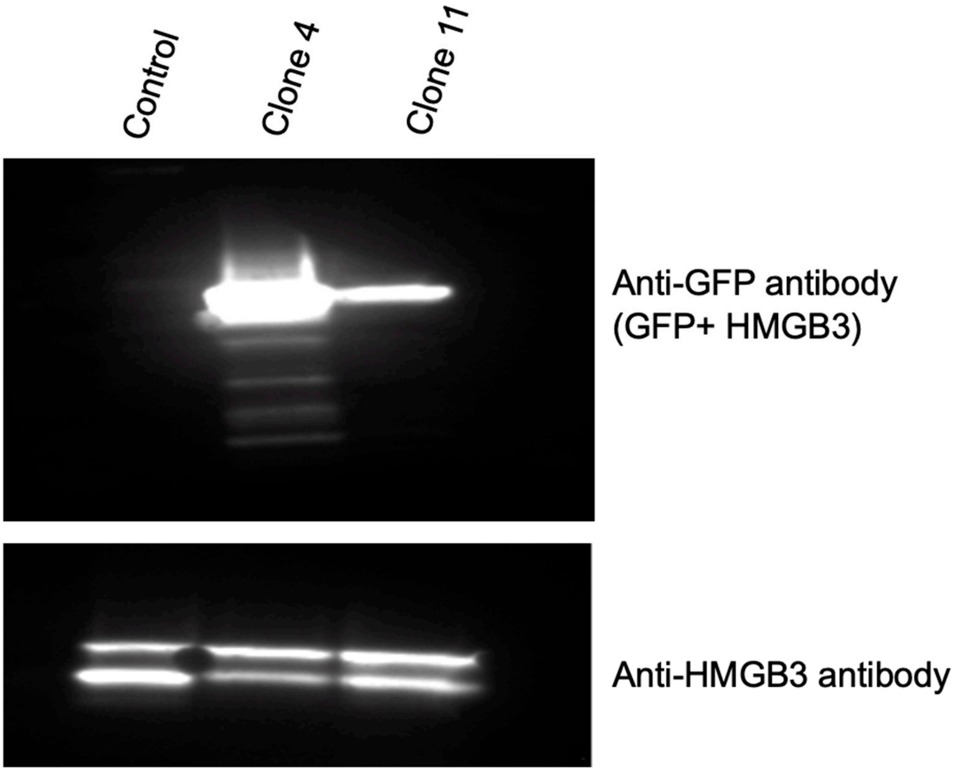

Figure S4: Assessment of GFP-tagged HMGB3 expression in human A2780 clones.

Table S1: Top differentially expressed genes that are upregulated in HMGB3 knockout clones compared to wild-type U2OS cells.

gene_name	padj	log2fc	fdrLvl	fcLvl	gene_type	description
MAP1A	3.88761365260241E-157	4.37401735701959	fdr_0.01	fc_2.up	protein_coding	microtubule associated protein 1A [Source:HGNC Symbol;Acc:HGNC:6835]
RBP1	4.84879566961486E-123	3.14053724518539	fdr_0.01	fc_2.up	protein_coding	retinol binding protein 1 [Source:HGNC Symbol;Acc:HGNC:9919]
NES	6.18276583141487E-120	2.2947265804009	fdr_0.01	fc_2.up	protein_coding	nestin [Source:HGNC Symbol;Acc:HGNC:7756]
MAGEA4	2.0898823282093E-90	2.47506741603108	fdr_0.01	fc_2.up	protein_coding	MAGE family member A4 [Source:HGNC Symbol;Acc:HGNC:6802]
KRT17	6.85038413428409E-69	2.30058452072471	fdr_0.01	fc_2.up	protein_coding	keratin 17 [Source:HGNC Symbol;Acc:HGNC:6427]
MYEOV	5.64186040490166E-63	3.5515590209431	fdr_0.01	fc_2.up	protein_coding	myeloma overexpressed [Source:HGNC Symbol;Acc:HGNC:7563]
ECM1	5.39384899651527E-58	3.48309893902573	fdr_0.01	fc_2.up	protein_coding	extracellular matrix protein 1 [Source:HGNC Symbol;Acc:HGNC:3153]
SSC4D	1.34959422205987E-55	3.20985615015575	fdr_0.01	fc_2.up	protein_coding	scavenger receptor cysteine rich family member with 4 domains [Source:HGNC Symbol;Acc:HGNC:14461]
PAGE2B	5.54603033703766E-55	3.24718165089691	fdr_0.01	fc_2.up	protein_coding	PAGE family member 2B [Source:HGNC Symbol;Acc:HGNC:31805]
COL1A1	4.17687858959459E-54	3.10262402528611	fdr_0.01	fc_2.up	protein_coding	collagen type I alpha 1 chain [Source:HGNC Symbol;Acc:HGNC:2197]
SERPINF1	1.66082856306943E-49	5.28834020121811	fdr_0.01	fc_2.up	protein_coding	serpin family F member 1 [Source:HGNC Symbol;Acc:HGNC:8824]
APOL1	2.91008158579892E-49	2.13526075438062	fdr_0.01	fc_2.up	protein_coding	apolipoprotein L1 [Source:HGNC Symbol;Acc:HGNC:618]
GPSM3	2.19296405434645E-47	2.910721793576	fdr_0.01	fc_2.up	protein_coding	G protein signaling modulator 3 [Source:HGNC Symbol;Acc:HGNC:13945]
MXRA8	5.53090931501732E-44	5.13080284576778	fdr_0.01	fc_2.up	protein_coding	matrix remodeling associated 8 [Source:HGNC Symbol;Acc:HGNC:7542]
MLLT11	2.06251092302973E-43	2.67217444996893	fdr_0.01	fc_2.up	protein_coding	MLLT11 transcription factor 7 cofactor [Source:HGNC Symbol;Acc:HGNC:16997]
IL32	1.06171326341018E-40	3.41390410446379	fdr_0.01	fc_2.up	protein_coding	interleukin 32 [Source:HGNC Symbol;Acc:HGNC:16830]
CA12	7.5941114496195E-39	2.44701831441133	fdr_0.01	fc_2.up	protein_coding	carbonic anhydrase 12 [Source:HGNC Symbol;Acc:HGNC:1371]
OLFML3	1.76429399178968E-37	2.15382546264423	fdr_0.01	fc_2.up	protein_coding	olfactomedin like 3 [Source:HGNC Symbol;Acc:HGNC:24956]
PLCB2	5.02607444976758E-37	4.8026469151442	fdr_0.01	fc_2.up	protein_coding	phospholipase C beta 2 [Source:HGNC Symbol;Acc:HGNC:9055]
HSD17B8	7.74643195280322E-37	3.30372451342013	fdr_0.01	fc_2.up	protein_coding	hydroxysteroid 17-beta dehydrogenase 8 [Source:HGNC Symbol;Acc:HGNC:3554]
LINC00973	1.05120898603457E-36	2.09349950779522	fdr_0.01	fc_2.up	lncRNA	long intergenic non-protein coding RNA 973 [Source:HGNC Symbol;Acc:HGNC:48868]
QPRT	2.69892257776639E-36	2.18839863629879	fdr_0.01	fc_2.up	protein_coding	quinolinate phosphoribosyltransferase [Source:HGNC Symbol;Acc:HGNC:9755]
HAPLN1	8.99911203645763E-35	2.23408643940592	fdr_0.01	fc_2.up	protein_coding	hyaluronan and proteoglycan link protein 1 [Source:HGNC Symbol;Acc:HGNC:2380]
SYT11	2.76538673188505E-34	2.32834235336103	fdr_0.01	fc_2.up	protein_coding	synaptotagmin 11 [Source:HGNC Symbol;Acc:HGNC:19239]
BHLHE41	4.26556196381298E-32	2.80662557215357	fdr_0.01	fc_2.up	protein_coding	basic helix-loop-helix family member e41 [Source:HGNC Symbol;Acc:HGNC:16617]
AQP1	1.86871722758681E-28	4.03406823951468	fdr_0.01	fc_2.up	protein_coding	aquaporin 1 (Colton blood group) [Source:HGNC Symbol;Acc:HGNC:633]
PARVG	1.27690388057695E-25	2.20072080871366	fdr_0.01	fc_2.up	protein_coding	parvin gamma [Source:HGNC Symbol;Acc:HGNC:14654]
PSMB9	8.06095487220978E-25	2.04041040393879	fdr_0.01	fc_2.up	protein_coding	proteasome 20S subunit beta 9 [Source:HGNC Symbol;Acc:HGNC:9546]
GPNMB	1.21937606229184E-24	3.46955866291023	fdr_0.01	fc_2.up	protein_coding	glycoprotein nmb [Source:HGNC Symbol;Acc:HGNC:4462]
PALM3	4.80289541941359E-24	5.50537754203565	fdr_0.01	fc_2.up	protein_coding	paralemmin 3 [Source:HGNC Symbol;Acc:HGNC:33274]
ANKRD20A9P	1.51370750761638E-23	2.91132186677715	fdr_0.01	fc_2.up	unprocessed_pseudogene	ankyrin repeat domain 20 family member A9, pseudogene [Source:HGNC Symbol;Acc:HGNC:42023]

gene_name	padj	log2fc	fdrLvl	fcLvl	gene_type	description
MAP1A	3.88761365260241E-157	4.374017357	fdr_0.01	fc_2.up	protein_coding	microtubule associated protein 1A [Source:HGNC Symbol;Acc:HGNC:6835]
RBP1	4.84879566961486E-123	3.140537245	fdr_0.01	fc_2.up	protein_coding	retinol binding protein 1 [Source:HGNC Symbol;Acc:HGNC:9919]
NES	6.18276583141487E-120	2.29472658	fdr_0.01	fc_2.up	protein_coding	nestin [Source:HGNC Symbol;Acc:HGNC:7756]
MAGEA4	2.0898823282093E-90	2.475067416	fdr_0.01	fc_2.up	protein_coding	MAGE family member A4 [Source:HGNC Symbol;Acc:HGNC:6802]
KRT17	6.85038413428409E-69	2.300584521	fdr_0.01	fc_2.up	protein_coding	keratin 17 [Source:HGNC Symbol;Acc:HGNC:6427]
MYEOV	5.64186040490166E-63	3.551559021	fdr_0.01	fc_2.up	protein_coding	myeloma overexpressed [Source:HGNC Symbol;Acc:HGNC:7563]
ECM1	5.39384899651527E-58	3.483098939	fdr_0.01	fc_2.up	protein_coding	extracellular matrix protein 1 [Source:HGNC Symbol;Acc:HGNC:3153]
SSC4D	1.34959422205987E-55	3.20985615	fdr_0.01	fc_2.up	protein_coding	scavenger receptor cysteine rich family member with 4 domains [Source:HGNC Symbol;Acc:HGNC:14461]
PAGE2B	5.54603033703766E-55	3.247181651	fdr_0.01	fc_2.up	protein_coding	PAGE family member 2B [Source:HGNC Symbol;Acc:HGNC:31805]
COL1A1	4.17687858959459E-54	3.102624025	fdr_0.01	fc_2.up	protein_coding	collagen type I alpha 1 chain [Source:HGNC Symbol;Acc:HGNC:2197]
SERPINF1	1.66082856306943E-49	5.288340201	fdr_0.01	fc_2.up	protein_coding	Serpin family F member 1 [Source:HGNC Symbol;Acc:HGNC:8824]
APOL1	2.91008158579892E-49	2.135260754	fdr_0.01	fc_2.up	protein_coding	apolipoprotein L1 [Source:HGNC Symbol;Acc:HGNC:618]
GPSM3	2.19296405434645E-47	2.910721794	fdr_0.01	fc_2.up	protein_coding	G protein signaling modulator 3 [Source:HGNC Symbol;Acc:HGNC:13945]

MXRA8	5.53090931501732E-44	5.130802846	fdr_0.01	fc_2.up	protein_coding	matrix remodeling associated 8 [Source:HGNC Symbol;Acc:HGNC:7542]
MLLT11	2.06251092302973E-43	2.67217445	fdr_0.01	fc_2.up	protein_coding	MLLT11 transcription factor 7 cofactor [Source:HGNC Symbol;Acc:HGNC:16997]
IL32	1.06171326341018E-40	3.413904104	fdr_0.01	fc_2.up	protein_coding	interleukin 32 [Source:HGNC Symbol;Acc:HGNC:16830]
CA12	7.5941114496195E-39	2.447018314	fdr_0.01	fc_2.up	protein_coding	carbonic anhydrase 12 [Source:HGNC Symbol;Acc:HGNC:1371]
OLFML3	1.76429399178968E-37	2.153825463	fdr_0.01	fc_2.up	protein_coding	Olfactomedin like 3 [Source:HGNC Symbol;Acc:HGNC:24956]
PLCB2	5.02607444976758E-37	4.802646915	fdr_0.01	fc_2.up	protein_coding	phospholipase C beta 2 [Source:HGNC Symbol;Acc:HGNC:9055]
HSD17B8	7.74643195280322E-37	3.303724513	fdr_0.01	fc_2.up	protein_coding	hydroxysteroid 17-beta dehydrogenase 8 [Source:HGNC Symbol;Acc:HGNC:3554]
LINC00973	1.05120898603457E-36	2.093499508	fdr_0.01	fc_2.up	lncRNA	long intergenic non-protein coding RNA 973 [Source:HGNC Symbol;Acc:HGNC:48868]
QPRT	2.69892257776639E-36	2.188398636	fdr_0.01	fc_2.up	protein_coding	quinolinate phosphoribosyltransferase [Source:HGNC Symbol;Acc:HGNC:9755]
HAPLN1	8.99911203645763E-35	2.234086439	fdr_0.01	fc_2.up	protein_coding	Hyaluronan and proteoglycan link protein 1 [Source:HGNC Symbol;Acc:HGNC:2380]
SYT11	2.76538673188505E-34	2.328342353	fdr_0.01	fc_2.up	protein_coding	synaptotagmin 11 [Source:HGNC Symbol;Acc:HGNC:19239]
BHLHE41	4.26556196381298E-32	2.806625572	fdr_0.01	fc_2.up	protein_coding	Basic helix-loop-helix family member e41 [Source:HGNC Symbol;Acc:HGNC:16617]
AQP1	1.86871722758681E-28	4.03406824	fdr_0.01	fc_2.up	protein_coding	aquaporin 1 (Colton blood group) [Source:HGNC Symbol;Acc:HGNC:633]
PARVG	1.27690388057695E-25	2.200720809	fdr_0.01	fc_2.up	protein_coding	parvin gamma [Source:HGNC Symbol;Acc:HGNC:14654]
PSMB9	8.06095487220978E-25	2.040410404	fdr_0.01	fc_2.up	protein_coding	Proteasome 20S subunit beta 9 [Source:HGNC Symbol;Acc:HGNC:9546]
GPNMB	1.21937606229184E-24	3.469558663	fdr_0.01	fc_2.up	protein_coding	glycoprotein nmb [Source:HGNC Symbol;Acc:HGNC:4462]
PALM3	4.80289541941359E-24	5.505377542	fdr_0.01	fc_2.up	protein_coding	paralemmin 3 [Source:HGNC Symbol;Acc:HGNC:33274]
ANKRD20A9P	1.51370750761638E-23	2.911321867	fdr_0.01	fc_2.up	unprocessed_ pseudogene	ankyrin repeat domain 20 family member A9, pseudogene [Source:HGNC Symbol;Acc:HGNC:42023]

Table S2: Top differentially expressed genes that are downregulated in HMGB3 knockout clones compared to wild-type U2OS cells.

gene_name	padj	log2fc	fdrLvl	fcLvl	gene_type	description
SARAF	5.91065757727107E-206	-2.06179913743188	fdr_0.01	fc_2.dn	protein_coding	store-operated calcium entry associated regulatory factor [Source:HGNC Symbol;Acc:HGNC:28789]
HMGB3	3.90470629924703E-170	-3.63167206166567	fdr_0.01	fc_2.dn	protein_coding	high mobility group box 3 [Source:HGNC Symbol;Acc:HGNC:5004]
ZNF395	3.85965420383006E-149	-2.25967704990934	fdr_0.01	fc_2.dn	protein_coding	zinc finger protein 395 [Source:HGNC Symbol;Acc:HGNC:18737]
DCTN6-DT	3.85965420383006E-149	-2.42422636852827	fdr_0.01	fc_2.dn	lncRNA	DCTN6 divergent transcript [Source:HGNC Symbol;Acc:HGNC:55241]
MAGEA10	1.1808043668958E-137	-7.67268609562714	fdr_0.01	fc_2.dn	protein_coding	MAGE family member A10 [Source:HGNC Symbol;Acc:HGNC:6797]
DCTN6	1.12787421019868E-100	-2.47205150888565	fdr_0.01	fc_2.dn	protein_coding	dynactin subunit 6 [Source:HGNC Symbol;Acc:HGNC:16964]
H2AC18	3.75115070025009E-100	-2.70117055905046	fdr_0.01	fc_2.dn	protein_coding	H2A clustered histone 18 [Source:HGNC Symbol;Acc:HGNC:4736]
ENSG00000290032	7.58825787337142E-89	-3.34468013559803	fdr_0.01	fc_2.dn	lncRNA	novel transcript
H2AC19	3.78466860910201E-85	-2.68055972373367	fdr_0.01	fc_2.dn	protein_coding	H2A clustered histone 19 [Source:HGNC Symbol;Acc:HGNC:29668]
FBXL13	4.96921112368377E-73	-3.51987744449834	fdr_0.01	fc_2.dn	protein_coding	F-box and leucine rich repeat protein 13 [Source:HGNC Symbol;Acc:HGNC:21658]
MAGI2-AS3	7.25190042546174E-72	-2.08819929451418	fdr_0.01	fc_2.dn	lncRNA	MAGI2 antisense RNA 3 [Source:HGNC Symbol;Acc:HGNC:40862]
H2BC11	3.67399286849844E-53	-2.57998372853585	fdr_0.01	fc_2.dn	protein_coding	H2B clustered histone 11 [Source:HGNC Symbol;Acc:HGNC:4761]
ATOH8	1.54428097716E-51	-3.48716722469501	fdr_0.01	fc_2.dn	protein_coding	atonal bHLH transcription factor 8 [Source:HGNC Symbol;Acc:HGNC:24126]
H2AC6	8.62885328310365E-51	-3.04148513844856	fdr_0.01	fc_2.dn	protein_coding	H2A clustered histone 6 [Source:HGNC Symbol;Acc:HGNC:4733]
ANXA10	8.74958862793373E-43	-8.64814999617499	fdr_0.01	fc_2.dn	protein_coding	annexin A10 [Source:HGNC Symbol;Acc:HGNC:534]
ZNF283	1.68070432806832E-42	-2.59591148644645	fdr_0.01	fc_2.dn	protein_coding	zinc finger protein 283 [Source:HGNC Symbol;Acc:HGNC:13077]
ENSG00000259607	3.94986294213989E-37	-2.0271162444187	fdr_0.01	fc_2.dn	lncRNA	novel transcript, antisense to KIF13B
GGTLC2	1.28281279919262E-34	-2.8235378228154	fdr_0.01	fc_2.dn	protein_coding	gamma-glutamyltransferase light chain 2 [Source:HGNC Symbol;Acc:HGNC:18596]
KIF13B	5.15037068305234E-32	-2.23996495190231	fdr_0.01	fc_2.dn	protein_coding	kinesin family member 13B [Source:HGNC Symbol;Acc:HGNC:14405]
GGTLC3	1.14872248803883E-31	-5.00726042892166	fdr_0.01	fc_2.dn	protein_coding	gamma-glutamyltransferase light chain family member 3 [Source:HGNC Symbol;Acc:HGNC:33426]
PAPPA-AS1	7.07033099049821E-30	-2.15485617890291	fdr_0.01	fc_2.dn	lncRNA	PAPPA antisense RNA 1 [Source:HGNC Symbol;Acc:HGNC:35152]
NXPH2	8.34189765759828E-24	-2.91119760816712	fdr_0.01	fc_2.dn	protein_coding	neurexophilin 2 [Source:HGNC Symbol;Acc:HGNC:8076]
CAVIN2	1.12181657374275E-22	-2.10825748166865	fdr_0.01	fc_2.dn	protein_coding	caveolae associated protein 2 [Source:HGNC Symbol;Acc:HGNC:10690]
LINC02331	1.32574774306044E-22	-2.67307157110833	fdr_0.01	fc_2.dn	lncRNA	long intergenic non-protein coding RNA 2331 [Source:HGNC Symbol;Acc:HGNC:53251]
H2BC4	1.91316349767522E-22	-2.15215371676817	fdr_0.01	fc_2.dn	protein_coding	H2B clustered histone 4 [Source:HGNC Symbol;Acc:HGNC:4757]
H4C8	2.53649015056812E-21	-2.3725254541163	fdr_0.01	fc_2.dn	protein_coding	H4 clustered histone 8 [Source:HGNC Symbol;Acc:HGNC:4788]
MSLN	3.16771881796222E-20	-2.05139104879532	fdr_0.01	fc_2.dn	protein_coding	mesothelin [Source:HGNC Symbol;Acc:HGNC:7371]
DYNLRB2-AS1	6.61566307376242E-20	-2.20393603771633	fdr_0.01	fc_2.dn	lncRNA	DYNLRB2 antisense RNA 1 [Source:HGNC Symbol;Acc:HGNC:55405]
H2BC12	1.70350223224157E-17	-2.44091223986247	fdr_0.01	fc_2.dn	protein_coding	H2B clustered histone 12 [Source:HGNC Symbol;Acc:HGNC:13954]

gene_name	padj	log2fc	fdrLvl	fcLvl	gene_type	description
SARAF	5.91065757727107E-206	-2.061799137	fdr_0.01	fc_2.dn	protein_coding	store-operated calcium entry associated regulatory factor [Source:HGNC Symbol;Acc:HGNC:28789]
HMGB3	3.90470629924703E-170	-3.631672062	fdr_0.01	fc_2.dn	protein_coding	high mobility group box 3 [Source:HGNC Symbol;Acc:HGNC:5004]
ZNF395	3.85965420383006E-149	-2.25967705	fdr_0.01	fc_2.dn	protein_coding	zinc finger protein 395 [Source:HGNC Symbol;Acc:HGNC:18737]
DCTN6-DT	3.85965420383006E-149	-2.424226369	fdr_0.01	fc_2.dn	lncRNA	DCTN6 divergent transcript [Source:HGNC Symbol;Acc:HGNC:55241]
MAGEA10	1.18080436689 58E-137	-7.672686096	fdr_0.01	fc_2.dn	protein_coding	MAGE family member A10 [Source:HGNC Symbol;Acc:HGNC:6797]
DCTN6	1.12787421019868E-100	-2.472051509	fdr_0.01	fc_2.dn	protein_coding	dynactin subunit 6 [Source:HGNC Symbol;Acc:HGNC:16964]
H2AC18	3.75115070025009E-100	-2.701170559	fdr_0.01	fc_2.dn	protein_coding	H2A clustered histone 18 [Source:HGNC Symbol;Acc:HGNC:4736]
ENSG00000290032	7.58825787337142E-89	-3.344680136	fdr_0.01	fc_2.dn	lncRNA	novel transcript
H2AC19	3.78466860910201E-85	-2.680559724	fdr_0.01	fc_2.dn	protein_coding	H2A clustered histone 19 [Source:HGNC Symbol;Acc:HGNC:29668]
FBXL13	4.96921112368377E-73	-3.519877444	fdr_0.01	fc_2.dn	protein_coding	F-box and leucine rich repeat protein 13 [Source:HGNC Symbol;Acc:HGNC:21658]
MAGI2-AS3	7.25190042546174E-72	-2.088199295	fdr_0.01	fc_2.dn	lncRNA	MAGI2 antisense RNA 3 [Source:HGNC Symbol;Acc:HGNC:40862]
H2BC11	3.67399286849844E-53	-2.579983729	fdr_0.01	fc_2.dn	protein_coding	H2B clustered histone 11 [Source:HGNC Symbol;Acc:HGNC:4761]
ATOH8	1.54428097716E-51	-3.487167225	fdr_0.01	fc_2.dn	protein_coding	atonal bHLH transcription factor 8 [Source:HGNC Symbol;Acc:HGNC:24126]
H2AC6	8.62885328310365E-51	-3.041485138	fdr_0.01	fc_2.dn	protein_coding	H2A clustered histone 6 [Source:HGNC Symbol;Acc:HGNC:4733]
ANXA10	8.74958862793373E-43	-8.648149996	fdr_0.01	fc_2.dn	protein_coding	annexin A10 [Source:HGNC Symbol;Acc:HGNC:534]
ZNF283	1.68070432806832E-42	-2.595911486	fdr_0.01	fc_2.dn	protein_coding	Zinc finger protein 283 [Source:HGNC Symbol;Acc:HGNC:13077]
ENSG00000259607	3.94986294213989E-37	-2.027116244	fdr_0.01	fc_2.dn	lncRNA	novel transcript, antisense to KIF13B

GGTLC2	1.28281279919262E-34	-2.823537823	fdr_0.01	fc_2.dn	protein_coding	gamma-glutamyltransferase light chain 2 [Source:HGNC Symbol;Acc:HGNC:18596]
KIF13B	5.15037068305234E-32	-2.239964952	fdr_0.01	fc_2.dn	protein_coding	kinesin family member 13B [Source:HGNC Symbol;Acc:HGNC:14405]
GGTLC3	1.14872248803883E-31	-5.007260429	fdr_0.01	fc_2.dn	protein_coding	gamma-glutamyltransferase light chain family member 3 [Source:HGNC Symbol;Acc:HGNC:33426]
PAPPA-AS1	7.07033099049821E-30	-2.154856179	fdr_0.01	fc_2.dn	lncRNA	PAPPA antisense RNA 1 [Source:HGNC Symbol;Acc:HGNC:35152]
NXPH2	8.34189765759828E-24	-2.911197608	fdr_0.01	fc_2.dn	protein_coding	neurexophilin 2 [Source:HGNC Symbol;Acc:HGNC:8076]
CAVIN2	1.12181657374275E-22	-2.108257482	fdr_0.01	fc_2.dn	protein_coding	caveolae associated protein 2 [Source:HGNC Symbol;Acc:HGNC:10690]
LINC02331	1.32574774306044E-22	-2.673071571	fdr_0.01	fc_2.dn	lncRNA	long intergenic non-protein coding RNA 2331 [Source:HGNC Symbol;Acc:HGNC:53251]
H2BC4	1.91316349767522E-22	-2.152153717	fdr_0.01	fc_2.dn	protein_coding	H2B clustered histone 4 [Source:HGNC Symbol;Acc:HGNC:4757]
H4C8	2.53649015056812E-21	-2.372525454	fdr_0.01	fc_2.dn	protein_coding	H4 clustered histone 8 [Source:HGNC Symbol;Acc:HGNC:4788]
MSLN	3.16771881796222E-20	-2.051391049	fdr_0.01	fc_2.dn	protein_coding	mesothelin [Source:HGNC Symbol;Acc:HGNC:7371]
DYNLRB2-AS1	6.61566307376242E-20	-2.203936038	fdr_0.01	fc_2.dn	lncRNA	DYNLRB2 antisense RNA 1 [Source:HGNC Symbol;Acc:HGNC:55405]
H2BC12	1.70350223224157E-17	-2.44091224	fdr_0.01	fc_2.dn	protein_coding	H2B clustered histone 12 [Source:HGNC Symbol;Acc:HGNC:13954]

SI References

1. Mukherjee, A. and K.M. Vasquez, *HMGB1 interacts with XPA to facilitate the processing of DNA interstrand crosslinks in human cells.* Nucleic acids research, 2016. **44**(3): p. 1151-1160.
2. Balana, A.T., et al., *O-GlcNAcylation of high mobility group box 1 (HMGB1) alters its DNA binding and DNA damage processing activities.* Journal of the American Chemical Society, 2021. **143**(39): p. 16030-16040.
